# Phenolic Fingerprinting and Bioactivity Profiling of Extracts and Isolated Compounds from *Gypothamnium pinifolium* Phil.

**DOI:** 10.3390/antiox11122313

**Published:** 2022-11-22

**Authors:** Ruth E. Barrientos, Elena Ibáñez, Adrián Puerta, José M. Padrón, Adrián Paredes, Fredi Cifuentes, Javier Romero-Parra, Javier Palacios, Jorge Bórquez, Mario J. Simirgiotis

**Affiliations:** 1 Instituto de Farmacia, Facultad de Ciencias, Universidad Austral de Chile, Campus Isla Teja, Valdivia 5090000, Chile; 2 Laboratory of Foodomics, Institute of Food Science Research, CIAL, CSIC, Nicolás Cabrera 9, 28049 Madrid, Spain; 3 BioLab, Instituto Universitario de Bio-Orgánica Antonio González (IUBO-AG), Universidad de La Laguna, 38206 La Laguna, Spain; 4 Laboratorio de Química Biológica, Instituto Antofagasta, Universidad de Antofagasta, Antofagasta 1270300, Chile; 5 Departamento de Química, Facultad de Ciencias Básicas, Universidad de Antofagasta, Antofagasta 1240000, Chile; 6 Laboratorio de Fisiología Experimental, Instituto Antofagasta, Universidad de Antofagasta, Antofagasta 1270300, Chile; 7 Departamento Biomédico, Facultad Ciencias de la Salud, Universidad de Antofagasta, Antofagasta 1240000, Chile; 8 Departamento de Química Orgánica y Fisicoquímica, Facultad de Ciencias Químicas y Farmacéuticas, Universidad de Chile, Olivos 1007, Casilla 233, Santiago 6640022, Chile; 9 Laboratorio de Bioquímica Aplicada, Química y Farmacia, Facultad de Ciencias de la Salud, Universidad Arturo Prat, Iquique 1110939, Chile

**Keywords:** *Gypothamnium*, phenolics, enzyme inhibition, native plants, antioxidant, coumarins, terpenes, hypotensive effects

## Abstract

*Gypothamnium pinifolium* Phil. (Asteraceae) is a small shrub that grows in the Paposo Valley of the II Antofagasta Region of Chile. This initial study is of the high-resolution phenolic fingerprinting, antioxidant activity, the relaxation effects in rat aorta, the inhibitory enzyme potential, plus the antiproliferative activity of the ethyl acetate and *n*-hexane extract from *G. pinifolium* and its two major isolated secondary metabolites (one coumarin: 2-*nor*-1,2-secolycoserone, and one diterpene: *ent*-labda-8,13-*E*-diene-15-ol). The study involves using ultra-high-performance liquid chromatography todiode array detection coupled with Q-Orbitrap mass spectrometry analysis (UHPLC-PDA-Orbi-trap-MS), in which various compounds were identified, including specific coumarins. The *n*-hexane extract showed total phenolic and flavonoid contents of 517.4 ± 12.5 mg GAE/100 g extract and 72.3 ± 3.7 mg QE/100 g extract, respectively. In addition, the antioxidant activity of the *n*-hexane extract was assessed using in-vitro assays such as bleaching of DPPH and ABTS (IC_50_: 14.3 ± 0.52 and 2.51 ± 0.43 µg extract/mL, respectively), FRAP (347.12 ± 1.15 μmol Trolox equivalent/g extract), and ORAC (287.3 ± 1.54 μmol Trolox equivalents/g extract). Furthermore, the inhibition against cholinesterases (acetylcholinesterase (AChE) 4.58 ± 0.04 µg/mL, butyrylcholinesterase (BChE) IC_50_: 23.44 ± 0.03 µg/mL) and tyrosinase (IC_50_: 9.25 ± 0.15 µg/mL) enzymes of the *n*-hexane extract, and main compounds (IC_50_: 1.21 ± 0.03 µg/mL, 11.23 ± 0.02 µg/mL, 3.23 ± 0.12 µg/mL, and 103.43 ± 16.86 µg/mL, correspondingly for the most active coumarin 1) were measured. The antiproliferative potential of the extracts and the two principal compounds against several solid human cancer cells was investigated. All of them showed good activity against cancer cells. Label-free live-cell imaging studies on HeLa cells exposed to the isolated coumarin and the diterpene enabled the observation of cell death and several apoptotic hallmarks. Our results indicate that *G. pinifolium* Phil. is a valuable source of secondary metabolites with potential activity against noncommunicable diseases.

## 1. Introduction

In recent years, the use of extracts rich in phenolic compounds from native shrubs or plants has been increasing due to their potential to ameliorate or support the treatment of noncommunicable or chronic diseases (NCDs). The main types include cardiovascular disease, cancer, chronic respiratory disease, and diabetes (WHO) [1]. These diseases, along with neurodegenerative diseases (Alzheimer’s and Parkinson’s), are the main causes of premature deaths and disability in the world, and consequently, approaches to the prevention of these diseases have become a main concern in the previous years [1,2].

Mitochondrial alterations, inflammation, plus oxidative stress, are molecular mechanisms that perform a central role in the initiation and progression of various NCDs [3]. Cardiovascular diseases account for most NCD deaths, and the incidence of cardiovascular events increases with age due to increased plasma cholesterol, increased arterial stiffness, and increased peripheral vascular resistance [4]. Furthermore, related to hypertension, extensive evidence from animal studies has provided convincing information about the impact of oxidative stress and inflammation on its genesis [5]. On the other hand, reactive oxygen species increase susceptibility to neuronal damage and functional impairment through brain oxidation in Alzheimer’s disease (AD), Parkinson’s disease (PD), and other neurodegenerative pathologies [6]. Recently, to fight the loss of neurotransmitters in Alzheimer’s disease, the approach of ligands directed at multiple targets and combined cholinesterase and monoamine oxidases inhibitors have been used. However, Alzheimer’s disease has become one of the greatest devastating pathologies owing to the absence of successful therapies [7].

Natural products represent a family of diverse molecules with a large range of bioactivities reported [8]. It has been uncovered that most antioxidant compounds can produce anti-inflammatory effects and that NCDs involve the overproduction of oxidants and oxidative damage of lipids, DNA, and proteins, so antioxidant phytochemicals might be crucial for their prevention and treatment [9].

Some phenolics from Chilean plants proved to be promising in the prevention or treatment of NCDs; for instance, our group has previously studied the Goji fruits and the dockings results for the main phenolic compound, chlorogenic acid, showed good fitting by calculations in the catalytic site of butyrylcholinesterase, glucosidase and α-amylase enzymes [10]. Moreover, our research team has studied many Chilean endemic plants of our interest, mainly for their polar phenolic compounds and their enzymatic inhibitory potential. For instance, from *Weinmannia trichosperma* cav. it was possible to perform the study of its main component, isoastilbin [11], a glycosylated flavonoid with a potent enzymatic inhibitory activity. Paposo Valley is on the coast of the Atacama Desert in the II Antofagasta Region of Chile. In this green spot, the endemic shrub *Gypothamnium pinifolium* Phil. grows freely: this is a smelly plant and producer of interesting coumarins as secondary metabolites [12]. To date, there is a unique scientific report concerning antiproliferative activity on breast cancer cell line MCF-7 from humans (ATCC), but no reports about the hypotensive and enzyme inhibition potential of coumarins from this plant have been published [12]. This study aimed to comprehensively perform a phytochemical screening of *G. pinifolium* crude extracts for its potential use in the food and pharmaceutical industries.

For the first time, we report the UHPLC-MS analysis of *G. pinifolium* extract with the aim of obtaining the phenolic fingerprint. We also include the measurements of antioxidant properties and hypotensive effects. Additionally, we report the potential for inhibition of cholinesterases and tyrosinase enzymes with docking experiments. Moreover, we evaluated the antiproliferative activities of *G. pinifolium* for the first time, including an evaluation of the two major isolated constituents, the coumarin 2-*nor*-1,2-secolycoserone, and the diterpene *ent*-labda-8,13-*E*-diene-15-ol.

## 2. Materials and Methods

### 2.1. Chemicals

The systems Arium 126 61316-RO and Arium 611 UV unit (Sartorius, Goettingen, Germany) were employed to get the ultra-pure water. Folin–Ciocalteu’s phenol reagent, 2,4,6-tri(2-pyridyl)-s-triazine aluminum chloride, iron (III) chloride hexahydrate, 2,2′-azinobis(3-ethylbenothiazoline-6-sulfonic acid) diammonium salt, 2,2-Diphenyl-1-(2,4,6-trinitrophenyl) hydrazyl, (±)-6-Hydroxy-2,5,7,8-tetramethylchromane-2-carboxylic acid (purity > 97%), quercetin (purity > 97%), gallic acid (purity > 98%), dimethyl sulfoxide (DMSO), acetylcholinesterase from *Electrophorus electricus*, butyrylcholinesterase from equine serum, tyrosinase (from mushroom), levodopa, 2-hydroxymethyl-5-hydroxy-γ-pyrone, trichloroacetic acid (TCA, Merck, Darmstadt, Germany), fetal calf serum (Gibco, Grand Island, NY, USA), L-glutamine (Merck, Darmstadt, Germany), penicillin G (Sigma, St. Louis, MO, USA), streptomycin (Sigma, St. Louis, MO, USA) and sulforhodamine B (Sigma, St. Louis, MO, USA) were acquired from Sigma-Aldrich Chem. Co. (St. Louis, MO, USA) and extrasynthèse (Genay, France). Formic acid, methanol, ethyl acetate, and *n*-hexane of HPLC grade were obtained from Merk^®^ (Santiago, Chile).

### 2.2. Plant Material

*G. pinifolium* was collected in November 2019 by hand in the Paposo Valley, II Región de Antofagasta, Chile, and was authenticated by Jorge Macaya, a botanist from the University of Chile, Santiago, Chile. The voucher specimen GP-01152019 was left in the Laboratory of Natural Products of the Universidad Austral de Chile, Chile. Once the plant material was collected, cleaned, and then dried per 24 h at 37 °C in a forced air-drying oven (BOV-V125F, Biobase, Jinan, Shandong, China), then the aerial parts were grounded to powder in a mill (Milly50, Imbriano Macchine Agricole, Pianopantano, Italy).

### 2.3. Extraction and Isolation Procedure

We extracted 500 g of dried and grounded *G. pinifolium* three times with 1 L of *n*-hexane employing a Biobase ultrasonic bath UC-60A at room temperature for half an hour protected from light. The filtered extract was disposed of in a rotary evaporator at 330 mbar and 36 °C to obtain 34 g of crude *n*-hexane extract. Moreover, 500 g of sample was extracted three times with ethyl acetate at 240 mbar and 36 °C to obtain 25 g of extract. The isolation and purification of the two major compounds, 2-nor-1,2-secolycoserone and ent-labda-8,13-E-diene-15-ol from *G. pinifolium* Phil., was performed following the protocol described in the supplementary material.

### 2.4. UHPLC–DAD–MS Instrument

For the UHPLC-MS analysis of *G. pinifolium* extracts, an Ultimate 3000 system with a diode array detector hyphenated with a Thermo Q-Exactive MS focus mass spectrometer (Thermo, Bremen, Germany) was employed. For the sample preparation, 5 mg of extract was dissolved in 2 mL of methanol and filtered with a micropore membrane filter (PTFE, 0.45 µm), and the injection volume was 10 µL [13]. The data was acquired with Chromeleon 7.2 software and analyzed with Thermo XCalibur 3.1 Software. The LC parameters and MS parameters are described in the supplementary material.

### 2.5. Total Phenolic (TPC) and Total Flavonoid (TFC) Content

The TPC of *G. pinifolium* was measured employing the Folin–Ciocalteu reagent method, and the results are expressed as mg gallic acid equivalents/100 g of the dry extract [14]. On the other hand, the TFC of *G. pinifolium* was determined by the aluminum chloride method, and the results are expressed as mg of quercetin equivalents/100 g of the dry extract [15]. The measurements were done in triplicate, and the data is reported as the mean ± SD.

### 2.6. Antioxidant Activity

#### 2.6.1. Radical DPPH Inhibition

The radical DPPH inhibition was performed by mixing thoroughly 150 µL of DPPH 400 μM with 50 µL of the sample or positive control, the 96-well microplate was kept protected from the light, and after half hour of reaction, the absorbance was recorded at 515 nm in a Synergy HTX microplate reader [13,16]. All the measurements were performed in triplicate. The data are expressed as IC_50_ in µg of extract or standard per mL and reported as the mean ± SD. The determination of the IC_50_ was performed using linear regression. Methanol was employed as a negative control and BHT as a positive control. The percentage of DPPH radical inhibition was calculated according to the following equation:

$$Percentage\;of\;radical\;inhibition\;\left(\%\right)=\left(1-\frac{A-As}{ADPPH}\right)\times 100$$

*A*: is the absorbance of the mixture of extract and radical, *As*: is the absorbance of the methanol and extract, and *ADDPH*: is the absorbance of the radical work solution.

#### 2.6.2. ABTS^•+^ Scavenging Capacity

The ABTS^•+^ radical scavenging assay was employed to determine the antioxidant activity of *G. pinifolium* conventional extracts [11,17]; the radical solution was prepared 16 h before the measurements mixing 7 mM ABTS and 2.45 mM K_2_S_2_O_8_, and the solution was stored protected from the light at room temperature until the ABTS radical formation. For the assay, were mixed 250 µL of ABTS radical with an increasing concentration of sample or positive control (Trolox), and after half an hour, the absorbance was measured at 734 nm with a Synergy HTX microplate reader. The results are expressed as IC_50_ in µg of extract or Trolox per mL. The percentage of radical inhibition formula was employed for the ABTS inhibition calculation, then the IC_50_ was calculated through linear regression (concentration vs. inhibition percentage), and the data is reported as the mean ± SD.

#### 2.6.3. Ferric-Reducing Antioxidant Power Assay (FRAP)

The method of Ferric-Reducing Antioxidant Power Assay (FRAP) was employed [11], and the results from *G. pinifolium* were interpolated in a linear regression of Trolox to express the results as μmol Trolox equivalent per g of dry extract. For the reaction were mixed 10 µL of the sample with 290 µL of FRAP solution, and the absorbance was measured at 593 nm after 5 min of incubation. The assay was done in triplicate, and the results are reported as the mean ± SD.

#### 2.6.4. Reactive Oxygen Species (ROS) Scavenging Capacity

The oxygen radical absorbance capacity (ORAC) method was performed as previously described [18]. A Trolox curve was used to perform the quantification. The results were obtained by a regression equation between the sample concentration or Trolox and the area under the fluorescence decay curves. The results are expressed as in μmol Trolox equivalents per gram of dry extract. The ROS inhibition was performed in triplicate for each sample, and the values are reported as the mean ± SD.

### 2.7. Animals

The investigation was conducted according to the local ethics research committee of Universidad de Antofagasta, which ratified the experimental procedures (CEIC #275/2020 and CEIC #366/2022) for the use of a normotensive animal model (*n* = 3–6). The characteristics of the animals were as follows: male Sprague Dawley rats, six to eight weeks old, weighing between 170 and 200 g. The animals were kept at room temperature (22–25 °C) with 45–51% of humidity with ad libitum free-choice access to tap water and food and were randomized.

### 2.8. Isolation of Rat Aorta and Vascular Reactivity Assays

All the animals were euthanized by cervical dislocation and immediately proceeded to dissection of the aorta. Then the vascular tissue was arranged in organ baths that were filled with Krebs-Ringer bicarbonate solution pH 7.4 and remained at 37 °C with a constant flux of gas (95% O_2_ and 5% CO_2_). Once the tissue was stabilized and the integrity of the vascular endothelium was evaluated, the aortic rings were pre-contracted with 10^−6^ M phenylephrine (PE), and after ten minutes, increasing concentrations of *G. pinifolium* extract, 2-*nor*-1,2-secolycoserone or *ent*-labda-8,13-*E*-diene-15-ol were added in the organ bath.

### 2.9. Cholinesterase Inhibition

The Ellman method [16] was used for the determination of the inhibitory potential against AChE and BChE. The enzymes were dissolved in Tris-HCl buffer 50 mM at pH 8.0 at the final concentration of 0.26 U/mL, and samples were prepared at 2 mg/mL and dissolved in buffer. For the assay, 100 µL 5-dithio-bis (2-nitrobenzoic acid) (DTNB) 3 mM, 20 µL of the enzyme, 40 µL of buffer, 20 µL of sample or standard (galantamine). Finally, to start the reaction, we added 20 µL of the substrate (acetyl-thiocholine iodide for AChE inhibition or butyryl-thiocholine chloride for BChE inhibition assays), and the absorbance was recorded at 405 nm for half an hour at 37 °C. The analysis was performed in triplicate, and the results are expressed as IC_50_.

### 2.10. Tyrosinase Inhibition Assay

The dopachrome method [19] was employed for the evaluation of the enzymatic inhibitory potential of *G. pinifolium* against tyrosinase. The samples were dissolved in ethanol, and the tyrosinase was prepared in phosphate buffer (PBS) pH 6.8 at the concentration of 100 U/mL and levodopa at 2.5 mM. For the assay were used 96-wells microplate and 20 µL of extract or standard (kojic acid) were mixed with 30 µL of PBS, then were added 40 µL of tyrosinase and 40 µL of the substrate. Finally, after 15 min, the absorbance was recorded at 492 nm. The results are expressed as IC_50_.

### 2.11. Docking Studies

**Figure 1:**
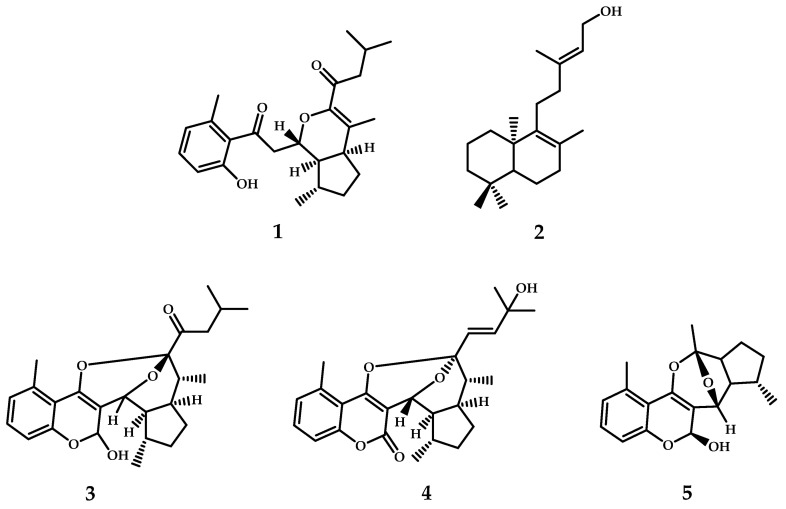
Compounds subjected to docking assays into the corresponding catalytic sites of acetylcholinesterase, butyrylcholinesterase, and tyrosinase: 2-*nor*-1,2-secolycoserone ((**1**), peak 26, Table 1), *ent*-labda-8,13-*E*-diene-15-ol (**2**), cyclolycoserone ((**3**), Peak 20, Table 1), 8-*epi*-gypothamniol ((**4**), peak 16, Table 1), 6-Hydroxyaphyllodenticulide ((**5**), peak 5, Table 1).

Docking simulations were completed for the main selected compounds obtained from the *G. pinifolium* extract and considering the proposed biosynthetic relationship between the coumarin derivatives detected (Figure 1). The docking studies are described in the supplementary material.

### 2.12. Statistical Analysis

The data acquired from vascular reactivity assays were expressed as average ± standard error of the mean (SEM). One-way analysis of variance (ANOVA) followed by the Dunnett test was employed for statistical data analysis. The determination of the half-maximal effective concentration (EC_50_) was performed using nonlinear regression (sigmoidal) via Graph Pad Prism software, version 8.0.1. Statistical significance is set at *p* = 0.05.

### 2.13. Antiproliferative Activity

Stock solutions in DMSO of the samples were prepared to dissolve the plant extract at a concentration of 100 mg/mL and the pure compounds **1**–**2** at 40 mM. For the antiproliferative activity tests, human solid tumor cells (100 µL) were seeded onto 96-well plates at a density of 2500 (A549, HBL-100, HeLa, and SW1573) or 5000 (T-47D, and WiDr) cells/well. 

On the next day, the samples were added to the cell cultures in triplicate at decimal dilutions in the range 250 to 2.5 µg/mL for the extracts and 100 to 0.001 µM for compounds **1**–**2**. Control cells were exposed to an equivalent amount of DMSO (0.25% *v*/*v*, negative control). Cells were incubated for an additional 48 h, after which time they were precipitated with 25 μL of ice-cold TCA (50% *w*/*v*) and fixed for 60 min at 4 °C [20]. Following sulforhodamine B staining, the optical density (OD) of each well was measured at 530 nm using a BioTek Power Wave XS absorbance microplate reader. Values were corrected with the background OD of the wells containing the control. The antiproliferative activity was expressed as a 50% reduction in cancer cell growth (GI_50_) and total growth inhibition (TGI). For compounds **1**–**2**, 50% lethal concentration (LC_50_) was also calculated. The cell lines used in this study were kindly provided by Prof. Godefridus J. Peters (VUmc, Amsterdam, The Netherlands).

### 2.14. Continuous Live Cell Imaging

The imaging platform microscope CX-A (Nanolive SA, Lausanne, Switzerland) was used to get refractive indexes (RIs), creating a holotomographic 3D image of the cells. HeLa cells were employed at a density of 50,000 cells/well onto an IBIDI μ-Dish, 35 mm high (IBIDI, Gräfelfing, Munich, Germany) and treated with compounds **1** and **2** right before the acquisition of the images at a dose of 80 µM and 50 µM, respectively. Data obtained were transferred to FIJI (NIH, Bethesda, MD, USA) for image analysis. The LIVE Cell Death Assay (LCDA) software (Nanolive SA) was used for the analysis of the RIs and for obtaining apoptosis kinetics.

## 3. Results and Discussion

### 3.1. Isolation and Structural Characterization of Secondary Metabolites

Compound isolation by adsorption chromatography performed for the *G. pinifolium*. *n*-hexane extract resulted in the structural characterization and identification of the two main compounds by nuclear magnetic resonance spectroscopy. After recrystallization at room temperature with an isocratic system composed of *n*-hexane: ethyl acetate 90:10, 532 mg of a pure compound was obtained in the form of colorless crystals, whose spectroscopic information is consistent with that of the coumarin 2-*nor*-1,2-secolycoserone [21] (**1**, Figure 1). Structure elucidated by ^1^H NMR (500 MHz, in CDCl_3_) and ^13^C NMR (100.13 MHz, in CDCl_3_): please see supplementary material [12].

Similarly, from fraction B, by medium pressure column chromatography (Kieselgel 60 G, and isocratic *n*-hexane: ethyl acetate 90:10 *v*:*v*), 35 mg of a pure compound was obtained in the form of colorless oil, whose spectroscopic information is consistent with that of the diterpene *ent*-labda-8,13-*E*-diene-15-ol (**2**, Figure 1). Structure elucidated by ^1^H NMR (500 MHz, in CDCl_3_) and ^13^C NMR (100.13 MHz, in CDCl_3_): please see supplementary material [12].

### 3.2. UHPLC–MS Analysis of G. pinifolium Extracts

**Figure 2:**
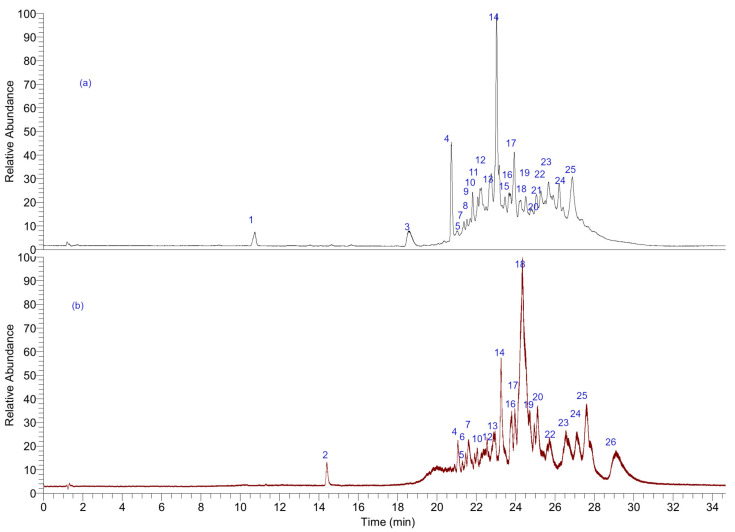
UHPLC-DAD chromatogram of *G. pinifolium* Phil. (**a**) ethyl acetate extract (**b**) *n*-hexane extract. The peak numbers correspond to those identified in Table 1.

The fingerprint analysis by UHPLC MS (Figure 2) of the crude extracts of *G. pinifolium* was investigated through high-resolution mass spectrometric analysis. A negative mode of detection was used. Some of the metabolites identified are reported for the first time in this species. Twenty-six compounds were detected and tentatively identified based on UV absorption and HR-MS fragmentation patterns (Table 1). The detailed fingerprinting analysis is explained below.

#### 3.2.1. Phenolic Coumarins and Derivatives

**Figure 3:**
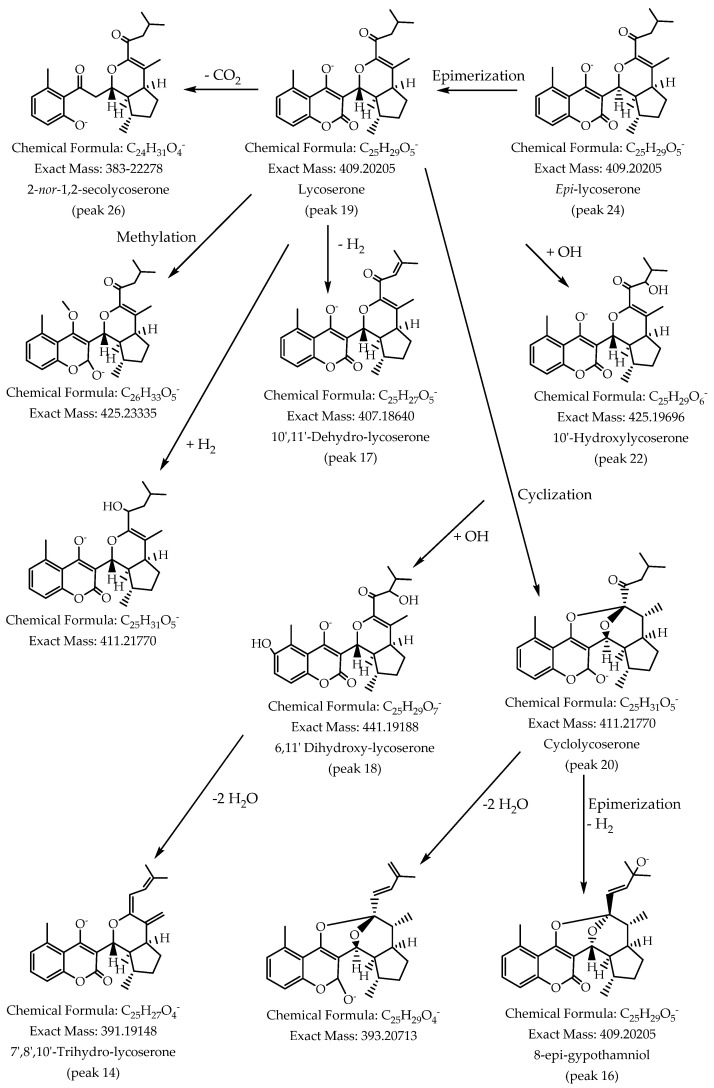
Proposed biosynthetic relationship between the coumarin derivatives detected in *G. pinifolium*.

Peak 1 was identified as diferulic acid (C_20_H_17_O_8_^−^), peak 2 with a [M-H]^−^ ion at m/z: 151.03960 was identified as vanillin (C_8_H_7_O_3_^−^), peak 9 with a parent ion at m/z: 343.15549 as the vanillyl derivative: 3,4-divanillyltetrahydrofuran (C_20_H_23_O_5_^−^), peak 3 as camphoric acid (C_10_H_15_O_4_^−^) and peak 4 as jacareubin (C_18_H_13_O_6_^−^) following, the isomer peaks 5 and 6 and 15 with pseudomolecular ions at m/z: 313.14478, 313.14490 and 313.14389, were identified as aphillodenticulide isomers [12]. Other peaks were identified as derivatives of lycoserone (peak 19, anion peak at m/z: 409.20245 C_25_H_29_O_5_^−^) isolated primarily from a *Lycoseris* species [22]. Derivatives’ presence can be explained through the biosynthetic relationship shown in Figure 3: Peak 24 was identified as the epimer at C-8′ (1′-α-H) of the latter compound (C_25_H_29_O_5_^−^) [23]. Peaks 20 and 21 were identified as cyclolycoserone and *epi*-cyclolycoserone [22] (C_25_H_31_O_5_^−^). In the same way, peaks 22 and 23 were identified as the isomers 10′-hydroxylycoserone and 11′-hydroxylycoserone, respectively (C_25_H_29_O_6_^−^). Peak 25 was identified as a reduced derivative (9-reduced-10′-11′ dehydro-lycoserone), peak 14 as 7′,8′,10′ trihydro-lycoserone (C_24_H_31_O_5_^−^), peak 17 as the dehydrogenated derivative 10′-11′ dehydro-lycoserone and peak 18 as the dihydroxy derivative: 6- 11′ dihydroxy-lycoserone (C_25_H_29_O_5_^−^). In the same manner, peak 26 was identified with spiking experiments with an authentic isolated compound as 2-*nor*-1,2-secolycoserone (C_24_H_31_O_4_^−^), [12], and peak 7 with a pseudomolecular ion at m/z: 415.21307 as the hydroxylated derivative 6, 12′ dihydroxy-2-*nor*-1,2-secolycoserone (C_24_H_31_O_6_^−^) and peak 13 as 12′ hydroxy-2-*nor*-1,2-secolycoserone (C_24_H_31_O_5_^−^). Peaks 11 and 16 were identified as gypothamniol and its epimer at C-8′(C_25_H_29_O_5_^−^), respectively (Figure 2) [23]. Finally, peak 8 with a [M-H]- ion at m/z: 357.13326 was identified as pinoresinol (C_20_H_21_O_6_^−^) [24].

#### 3.2.2. Terpenes

Peak 12 was identified by spiking experiments as *ent*-labda-8,13-*E*-diene-15-ol * (C_19_H_22_O_6_^−^) [12].

### 3.3. Total Phenolic and Flavonoid Contents and Antioxidant Activity

Results corresponding to total phenolic and flavonoid contents and antioxidant activity measured using the different in-vitro assays are summarized in Table 2 for *n*-hexane and ethyl acetate extracts of *G. pinifolium*. For the *n*-hexane and ethyl acetate extracts, the total phenolic content was 517.4 ± 12.5 and 538.4 ± 4.7 mg of gallic acid/100 g dry extract, respectively. Although the TPC results were similar between both extracts, for the total flavonoid content, the ethyl acetate extract obtained a higher result (465.8 ± 27.5 mg of quercetin/100 g dry extract), six times greater compared to the *n*-hexane extract (72.3 ± 3.7 mg of quercetin/100 g dry extract). For the antioxidant activity, the ethyl acetate extract also showed better results for DPPH, ABTS, FRAP, and ORAC assays compared with the *n*-hexane extract.

### 3.4. Vascular Relaxation Produced by G. pinifolium

**Figure 4:**
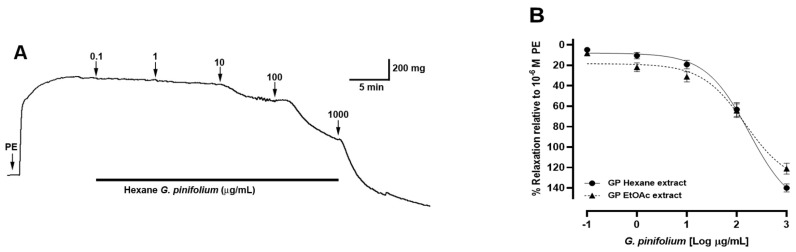
The relaxation effect of *G. pinifolium* in intact rat aorta. The rat aorta was pre-contracted with 10^−6^ M PE for 10 min, and then, rising concentrations of *G. pinifolium* Phil. (0.1 to 1000 µg/mL) were added in the organ bath each 7 min. The original record of the vascular effect of *n*-hexane extract of *G. pinifolium*. in intact rat aorta (**A**). Concentration-response curves for *G. pinifolium n*-hexane and ethyl acetate extract in intact aortic rings (**B**). Data are the average ± SEM of 6 independent experiments.

The vascular relaxation observed suggests that *G. pinifolium* would produce a potential hypotensive effect. The relaxation in the aorta (intact) with 100 μg/mL for *n*-hexane and ethyl acetate extract was 63 ± 7% and 64 ± 7%, respectively. Furthermore, with the highest concentration used of 1000 μg/mL, the relaxation was 140 ± 4% and 121 ± 5% for the *n*-hexane and ethyl acetate extract (Figure 4B). The half-maximal effective concentration (EC_50_) was not significantly different in the presence of *G. pinifolium n*-hexane extract (177 ± 1 μg/mL) or GP EtOAc extract in intact aortic rings (135 ± 1 μg/mL).

*G. pinifolium* extracts produce a good relaxation effect at 100 µg/mL in the pre-contracted intact aorta. Although the ethyl acetate extract showed higher antioxidant activity (FRAP, ABTS, and DPPH) than the *n*-hexane extract, the relaxation effect was similar in both extracts. Therefore, the biological activity should be due to other causes (synergy among the compounds, i.e., terpenoids and coumarins) and not by an increased antioxidant activity leading to increased bioavailability of the endothelial nitric oxide, which is an important vasodilator molecule.

**Figure 5:**
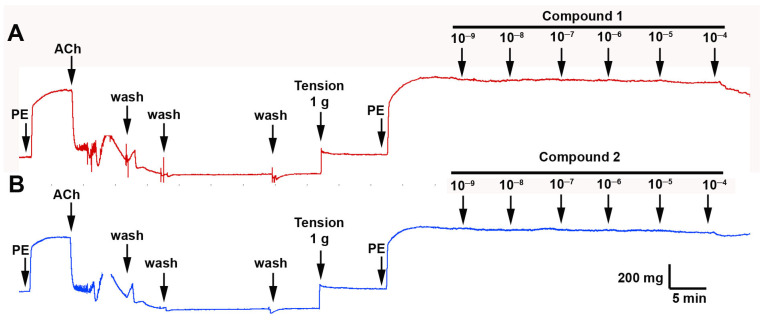
Compounds **1** and **2** do not cause vascular relaxation in the intact aorta. The original record of the vascular effect of compounds **1** (**A**) and **2** in rat aorta (**B**). The tissue was pre-contracted with 10^−6^ M PE, and then, the rising concentrations of compound **1** or **2** (10^−9^–10^−4^ M) were added to the bath. Three independent experiments were performed.

Compounds **1** and **2** showed a slight relaxation effect in aortic rings (Figure 5). Only at 10^−4^ M was the relaxation higher, but not significant. The effect between compounds **1** (coumarin derivative) and **2** (diterpenoid derivative) was not significantly different.

Natural products have been used as a source of new drugs, and it is possible that they will continue to play an important role in the search for lead compounds for the treatment of systemic arterial hypertension [25]. Coumarins are natural compounds known as benzopyrones. This group includes a large number of phenolic derivatives that have in common a benzene ring fused to an α-pyrone and depending on the different functional groups incorporated in their molecules, their bioactivity can change [26]. Natural coumarins such as khellactone derivatives and coumarin-chalcone fibrates reduce the increased calcium influx into cardiomyocytes, leading to an antihypertensive effect [27]. Other studies reported that isopentenyl residue in the coumarin backbone causes a reduction of calcium influx by blocking cation channels in vascular smooth muscle [28]. We believe that the vascular relaxation effect produced by *G. pinifolium* extract in the intact rat aorta may occur by a synergist effect among the compound **1** and **2** or that the extract may have another coumarin more potent is responsible for this activity because the percentage of relaxation effect of *G. pinifolium* extract (Figure 4) was greater than the effect of the isolated compound **1** or **2** (Figure 5). Moreover, diterpenoids have diverse biological activities and are an important group of natural products. Among the many medicinal plants known and studied for their antihypertensive activities, this class of molecules has been reported as the main compound [29]. Reports of forskolin, a labdane diterpenoid isolated from *Coleus forskohlii* Briq. (Lamiaceae) in different animal models have also been made. Through a vasodilator effect, it can reduce normal or high blood pressure, in addition to exerting a positive ionotropic action on the cardiac muscle, and can also produce a significant inhibition of platelet aggregation [30]. In addition, the 13-epi-9-deoxyforskolin, another labdane diterpenoid isolated from the same Indian medicinal plant, has shown antihypertensive activity in a previous study. An ability to reduce blood pressure was evaluated in anesthetized animals, demonstrating significant hypotensive activity [31]. It has been shown that *Marrubium vulgare* L. (Lamiaceae) contains marrubiin and marrubenol, compounds that are labdane diterpenes, in addition to an interesting mixture of phenolic compounds [32]. Moreover, it was found that these two diterpenes are capable of inhibiting aortic contraction through a concentration-dependent manner and that marrubenol was moderately more potent than marrubiin [33]; in another investigation with vascular tissue, the mechanism of the relaxant activity of marrubenol was studied, showing that this labdane diterpene is a potent inhibitor of the contraction evoked by potassium chloride 100 mM and demonstrating that the inhibition of the potassium chloride contraction of the vascular tissue was endothelium-independent [34]. In this work, the *ent*-labdane 2, *ent*-labda-8,13-*E*-diene-15-ol, showed no vascular relaxation in the intact aorta (Figure 5). Therefore, further studies involving more isolation steps of coumarins and diterpenoids and testing of minor constituents would be necessary to clarify this approach.

### 3.5. Enzymatic Inhibitory Activity

Due to the high content of phenolic compounds, the use of plants has been important over the years to prevent neurodegenerative diseases. Besides, most antioxidant phytochemicals have been found to have anti-inflammatory action and could play an important role in the prevention and treatment of chronic diseases that involve the overproduction of oxidants and oxidative damage to large biomolecules (lipids, DNA, and proteins) [9]. *G. pinifolium* extracts were assessed in vitro for cholinesterase and tyrosinase inhibitory potential. To the best of our knowledge, no previous reports regarding anti-enzymatic *potential have been conducted in this species.*

Previously, for other species from Chile, our group found good enzymatic inhibitions against AChE, BChE, and tyrosinase; in some cases, these results are similar to those obtained for *G. pinifolium*. The main results of the present study are summarized in Table 3 and are expressed as IC_50_ values (µg/mL). In the AChE inhibition assay, *G. pinifolium* showed an IC_50_ of 4.58 ± 0.04 µg/mL and 6.43 ± 0.03 µg/mL for *n*-hexane and ethyl acetate extracts, respectively, and for BChE inhibition assays the IC_50_ results were 23.44 ± 0.03 µg/mL and 33.25 ± 0.02 µg/mL for *n*-hexane and ethyl acetate extracts, respectively. On the other hand, in a similar way to *G. pinifolium*, the fruits (pulp and seeds) of *Greigia sphacelata* (Ruiz and Pav.) Regel (Bromeliaceae) showed potent inhibition of AChE for the pulp and seeds, the IC_50_ for AChE was 4.49 ± 0.08 µg/mL and 4.38 µg/mL, respectively [35]. Recently, for the ethanolic extract of *Himantormia lugubris* from Antarctica, a good inhibition for AChE with an IC_50_ of 12.38 µg/mL [36] was found. However, the AChE inhibitions produced by the *G. pinifolium* extracts used in this research are more potent. Also, for *Artemisia copa* Phil. (Asteraceae), ethanolic extract showed a potent anticholinesterase activity, with IC_50_ of 3.92 ± 0.08 µg/mL for AChE and 44.13 ± 0.10 µg/mL for BChE [16], for this last assay and in comparison with *G. pinifolium* extract the IC_50_ values showed a less potent activity, the same occurs if we compare the results for *G. sphacelata* for which the IC_50_ for pulp was 73.86 ± 0.09 µg/mL and for seeds 78.57 ± 0.06 µg/mL [35], and for the ethanolic extract of *H. lugubris* the enzyme inhibition against BChE was 31.54 ± 0.02 µg/mL, a value close to *G. pinifolium* ethyl acetate extract and less potent that *n*-hexane extract [36]. Likewise, for *Weinmannia trichosperma* Cav., the in vitro inhibitory effects against 5-hLOX, AChE, and BChE were investigated for the aqueous extract, and interesting activity as a 5-hLOX inhibitor was found. Additionally, the results for inhibitory activities for AChE and BChE were 3.13 ± 0.03 µg/mL and 2.94 ± 0.08 µg/mL, respectively [11].

Furthermore, for tyrosinase inhibition, *H. lugubris* showed moderate activity with an IC_50_ of 22.32 ± 0.21 µg/mL, and in comparison with the potent inhibition produced by *G. pinifolium n*-hexane and ethyl acetate extracts with IC_50_ values of 9.25 ± 0.15 µg/mL and 12.32 ± 0.21 µg/mL, respectively, showed a better capacity for inhibiting this enzyme [36]. Recently, for *Ovidia pillopillo* (Gay) Meissner (Thymelaeaceae) ethanolic extract, the IC_50_ value for tyrosinase inhibition was 9.92 ± 0.05 µg/mL, a potent and close result to that produced by *G. pinifolium n*-hexane extract.

These results confirm that *G. pinifolium* is an interesting source of compounds, phenolics, and specially coumarins, with the potential to inhibit enzymes related to NCDs, so this report highlights the importance of these metabolites for the prevention of neurodegenerative diseases.

For a better understanding of the results, molecular docking studies were performed, as discussed below.

### 3.6. Docking Studies

Some of the major identified metabolites and proposed biosynthetic epimeric derivatives were selected to test them as potential acetylcholinesterase, butyrylcholinesterase, and tyrosinase inhibitors by performing docking assays, according to the ultra-high performance liquid chromatography-photodiode array detection hyphenated with Orbitrap mass spectrometry analysis (UHPLC-PDA-Orbitrap-MS) of the *n*-hexane and ethyl acetate extracts from the *G. pinifolium* (Asteraceae). The latter allows us to get insights into the intermolecular interactions and energy descriptors between the derivatives and the corresponding enzyme catalytic sites, considering the inhibition assays obtained and shown in Table 3. Docking binding energies expressed in kcal/mol of each compound are shown in Table 4.

#### 3.6.1. Torpedo Californica Acetylcholinesterase (TcAChE) Docking Results

Binding energies shown in Table 4 indicate that every tested compound possesses a good profile as an acetylcholinesterase inhibitory agent, highlighting compound 2-*nor*-1,2-secolycoserone, which displayed binding energy of −13.396 kcal/mol. This result agrees with the experimental inhibition assays obtained and shown in Table 3, where both *n*-hexane and ethyl acetate extracts exhibited low half-maximal inhibitory concentration (IC_50_) values. Compared to galantamine, extracts’IC_50_ value is slightly higher, which could be explained by the more complex profile of extracts, with several active metabolites that can compete among them for the acetylcholinesterase catalytic site. This fact is supported by the similar IC_50_ range values of extracts compared to galantamine (Table 3) and by the inhibitory ability (Table 3) of isolated molecules 1 and 2, with IC_50_ values close to that of galantamine.

**Figure 6:**
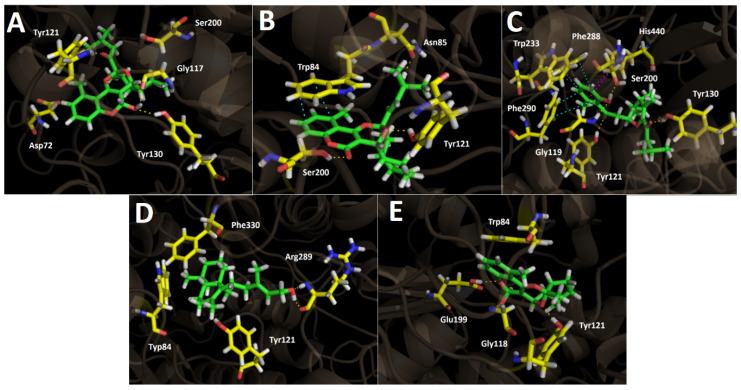
Predicted binding mode and predicted intermolecular interactions of selected major metabolites from the *Gypothamnium pinifolium n*-hexane and ethyl acetate extracts and the residues of *Torpedo Californica* acetylcholinesterase (*Tc*AChE) catalytic site. Yellow dotted lines indicate hydrogen bond interactions, cyan dotted lines represent π-π interactions, and magenta dotted lines represent T-shaped interactions. (**A**) Cyclolycoserone in the catalytic site; (**B**) 8-*epi*-gypothaminol in the catalytic site; (**C**) 2-*nor*-1,2-secolycoserone in the catalytic site; (**D**) *Ent*-labda-8,13-*E*-diene-15-ol in the catalytic site; (**E**) 6-hydroxyaphyllodenticulide in the catalytic site.

Docking results showed that cyclolycoserone interacts through three hydrogen bonds with the residues of Gly117 and Tyr130 (two of them through the hydroxyl group (–OH) at the 2*H*-chromene skeleton). The third hydrogen bond interaction occurs between the oxygen atom of the carbonyl group at the side chain of cyclolycoserone and the hydrogen atom (–H) of the –OH functional group of tyrosine (Figure 6A). Although 8-epi-Gypothaminol shows some structural similarities with cyclolycoserone (both possess an eight-membered ring with an acetal group), this compound arranges in the acetylcholinesterase catalytic site through three hydrogen bond interactions with Asn85, Tyr121, and Ser200, as well as an extra π-π interaction with the aromatic ring of Trp84 amino acid, which could be responsible for the best binding energy of −12.683 kcal/mol (Figure 6B).

The best binding energy of −13.396 kcal/mol, 2-*nor*-1,2-secolycoserone shows different (and more) interactions within the acetylcholinesterase catalytic site, such as four hydrogen bond interactions with Tyr121, Tyr130, Gly119, and Ser200. Moreover, this compound shows two π-π interactions with Phe288 and Phe290, but also two T-shaped interactions with the residues of Trp233 and His440, wherein the former interaction with the phenyl ring of 2-*nor*-1,2-secolycoserone produces the T tip of the interaction, while with His440 it is exactly the opposite, being the T tip the imidazole ring of this amino acid (Figure 6C).

*Ent*-labda-8,13-*E*-diene-15-ol, which exhibited an IC_50_ value of 5.45 ± 0.02 µg/mL, shows two hydrogen bond interactions with the amino acid of Arg289, whereas 6-hydroxyaphyllodenticulide, which is a 6-carbonyl reduced derivative of aphyllodenticulide, shows three hydrogen bond interactions through its different oxygenated groups: the oxygen atom of the 2*H*-chromene core and Glu199, the –OH function and Gly118 and one of the oxygen atoms of the dioxabicyclooctane and the residue of Tyr 121 (Figure 6E).

#### 3.6.2. Butyrylcholinesterase (hBChE) Docking Results

Binding energies from docking assays over butyrylcholinesterase of selected major compounds from the *G. pinifolium n*-hexane and ethyl acetate extracts showed good binding energies, suggesting that they could behave as good butyrylcholinesterase inhibitors. Although tested derivatives show slightly better energies compared to galantamine, no big differences are observed. Indeed, both extracts and molecules 1, 2, and 3 displayed similar order of IC_50_ values. Therefore, it would be reasonable to propose once again, as in acetylcholinesterase docking results, that the complex profile, including different metabolites in the extracts, competes for the butyrylcholinesterase catalytic site preventing lower IC_50_ values.

**Figure 7:**
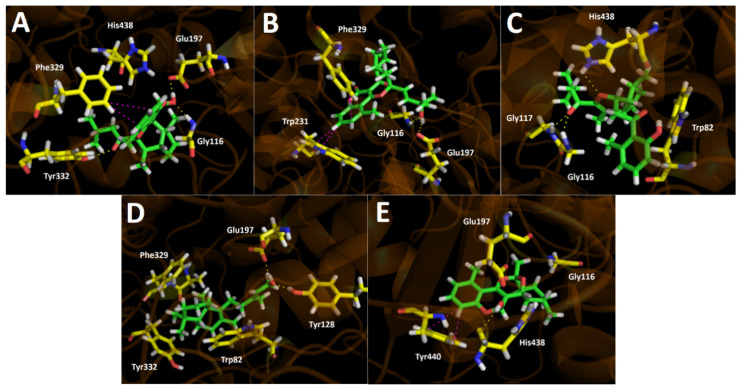
Predicted binding mode and predicted intermolecular interactions of selected major metabolites from the *Gypothamnium pinifolium n*-hexane and ethyl acetate extracts and the residues of human butyrylcholinesterase (*h*BChE) catalytic site. Yellow dotted lines indicate hydrogen bond interactions, and magenta dotted lines represent T-shaped interactions. (**A**) Cyclolycoserone in the catalytic site; (**B**) 8-epi-gypothaminol in the catalytic site; (**C**) 2-*nor*-1,2-secolycoserone in the catalytic site; (**D**) *Ent*-labda-8,13-*E*-diene-15-ol in the catalytic site; (**E**) 6-hydroxyaphyllodenticulide in the catalytic site.

Hydrogen bond interactions and T-shaped interactions for the selected major derivatives in the butyrylcholinesterase catalytic site were predominant. Cyclolycoserone interacts through three hydrogen bonds, two through the –OH at the 2*H*-chromene moiety and the residues of Gly116 and Glu197, and the third one between the oxygen atom of the carbonyl at the side chain of this derivative and Tyr332 exist. Likewise, a T-shaped interaction between this metabolite’s phenyl core and Phe329 amino acid can also be observed (Figure 7A). 8-epi-Gypothaminol arranged in the butyrylcholinesterase catalytic site in the opposite direction, allowing two hydrogen bond interactions through its only –OH functional group and the residues of Gly116 and Glu197, as well as two T-shaped interactions between the phenyl skeleton of the 2*H*-chromene core and Trp231 and Phe329 amino acids (Figure 7B).

Both 2-*nor*-1,2-secolycoserone and acetylcholinesterase docking assays showed the best binding energy descriptor (−9.738 kcal/mol), which could be attributed to the presence of three hydrogen bond interactions with Gly117, Gly116, and His438 through the oxygen atoms of the 3,4-dihydro-2H-pyran moiety and the carbonyl function of its side chain respectively (Figure 7C).

On the other hand, *ent*-labda-8,13-*E*-diene-15-ol and 6-hydroxyaphyllodenticulide displayed binding energies of −8.206 kcal/mol and −8.086 kcal/mol, respectively. Both showed the lowest energies, probably due to fewer hydrogen bond interactions and some hydrophobic interactions as well. *Ent*-labda-8,13-*E*-diene-15-ol shows two hydrogen bond interactions with Tyr128 and Glu197 through the only –OH group that this derivative contains (Figure 7D), while 6-hydroxyaphyllodenticulide shows the ability to interact through a hydrogen bond with His438 and the –OH function that the 2*H*-chromene skeleton bears, as well as T-shaped interaction between the phenyl moiety and Tyr440 (Figure 7E).

#### 3.6.3. Tyrosinase Docking Results

**Figure 8:**
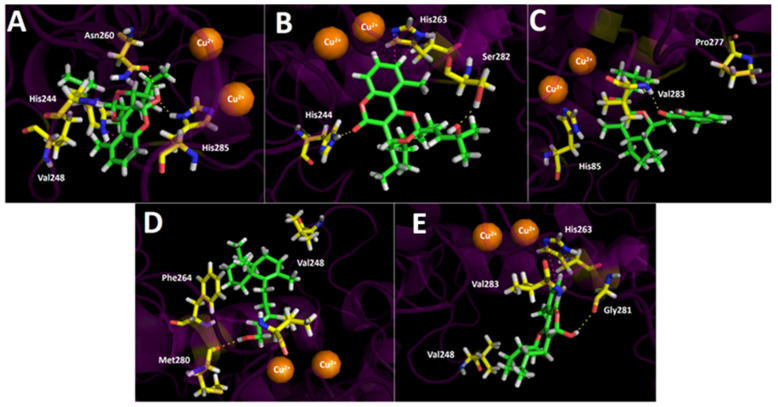
Predicted binding mode and predicted intermolecular interactions of selected major metabolites from the *Gypothamnium pinifolium n*-hexane and ethyl acetate extracts and the residues of *Agaricus bisporus* mushroom tyrosinase catalytic site yellow dotted lines indicate hydrogen bond interactions and magenta dotted lines represent T-shaped interactions. (**A**) Cyclolycoserone in the catalytic site; (**B**) 8-epi-gypothaminol in the catalytic site; (**C**) 2-*nor*-1,2-secolycoserone in the catalytic site; (**D**) *Ent*-labda-8,13-*E*-diene-15-ol in the catalytic site; (**E**) 6-hydroxyaphyllodenticulide in the catalytic site.

Inhibition assays of the selected major metabolites from the *G. pinifolium n*-hexane and ethyl acetate extracts over tyrosinase turned out to be close to those for acetylcholinesterase and butyrylcholinesterase. The latter results could be explained by our docking results, summarized in Table 4 and Figure 8.

The binding energies of selected major compounds obtained from the UHPLC chromatogram suggest that the main inhibitory activity would lie in 2-*nor*-1,2-secolycoserone over tyrosinase inhibition. As a matter of fact, the inhibitory experiments shown in Table 3 exhibited that the IC_50_ value for 2-*nor*-1,2-secolycoserone was 3.23 ± 0.12 µg/mL being both assays in total agreement.

Regarding the intermolecular interactions in the tyrosinase catalytic site, the main responsible for the enzyme inhibition would be hydrogen bond interactions and T-shaped interactions. Therefore, cyclolycoserone, which exhibits binding energy of −7.047 kcal/mol, shows two hydrogen bond interactions with Asn260 and His285 through its –OH at the 2*H*-chromene moiety (Figure 8A). 8-epi-gypothaminol, which possesses its 2*H*-chromene in an opposite arrangement in the tyrosinase catalytic site compared to cyclolycoserone, also interacts through two hydrogen bonds. Nonetheless, since 8-epi-gypothaminol fits differently, Ser282 and His244 are the amino acids involved in the hydrogen bond interactions, sharing the responsibility of the –OH function at the side chain and the carbonyl group (C=O) at the 2*H*-chromene skeleton respectively. Besides, 8-epi-gypothaminol performs a T-shaped interaction between His263 and its phenyl core (Figure 8B)

On the other hand, as can be seen in Figure 8C, 2-*nor*-1,2-secolycoserone shows interactions through one of its carbonyl groups (the main hydrogen bond interaction), as well as some hydrophobic interactions that allow for fitting properly in the tyrosinase catalytic site. In the same way, *ent*-labda-8,13-*E*-diene-15-ol (Figure 8D) also shows one hydrogen bond interaction through the hydrogen atom of the –OH functional group of its side chain and the carbonyl group of Met280 residue.

Finally, 6-hydroxyaphyllodenticulide, which bears a dioxabicyclooctane ring in its structure, once again showed restricted binding energy, suggesting that it is not a contributing inhibitor compared to those tested in our docking experiments and which are present in the *Gypothamnium pinifolium* extracts. Also, 6-hydroxyaphyllodenticulide can interact through two hydrogen bonds with Gly281 and Val283. Furthermore, a T-shaped interaction is also observed between the phenyl moiety and the imidazole aromatic ring of the amino acid His263 (Figure 8E).

### 3.7. Antiproliferative Activity

As part of our interest in the bioprospection of microorganisms [37,38,39] and native plants [40,41,42,43,44], we tested the antiproliferative activity of the *n*-hexane extract of *G. pinifolium* against a panel of six representative human solid tumor cell lines: A549 and SW1573 (lung), HBL-100 and T-47D (breast), HeLa (cervix) and WiDr (colon). The GI_50_ values showed growth inhibition in all cell lines in the range of 7.6–20 µg/mL. Accordingly, we analyzed the two main products contained in the extract. Thus, isolated compounds **1**–**2** were tested against the same cell panel. Results are shown in Table 5. The two products displayed growth inhibition against all the cell lines. Compound **2** (GI_50_ 4.5–10 µM) resulted in more active than **1** and with similar potency to the standard anticancer drug cisplatin. This result is consistent with a previous study, where compound **1** was reported to induce cell growth inhibition in the cancer line MCF-7 after 24 h of drug exposure (IC_50_ = 15 µM) [12].

According to Zhu, J.J. & Jiang [27], some coumarins can inhibit tumor angiogenesis [45] and serine proteases, down-regulate NF-kB, and induce caspase-dependent apoptosis through the mitochondrial pathway [46]. Therefore, further studies in this direction are necessary to identify the cellular target responsible for the biological activity of coumarin **1**. In the case of terpene **2**, the SwissTargetPrediction database (URL http://www.swisstargetprediction.ch/, accessed on 21 June 2022) provided a hint on predicted macromolecular targets. The analysis revealed low probability against all targets, which implies that compound **2** holds a novel structure as a bioactive substance. Thus, no initial hint is available, and the mechanism of action should be further studied from the beginning.

### 3.8. Continuous Live-Cell Imaging

**Figure 9:**
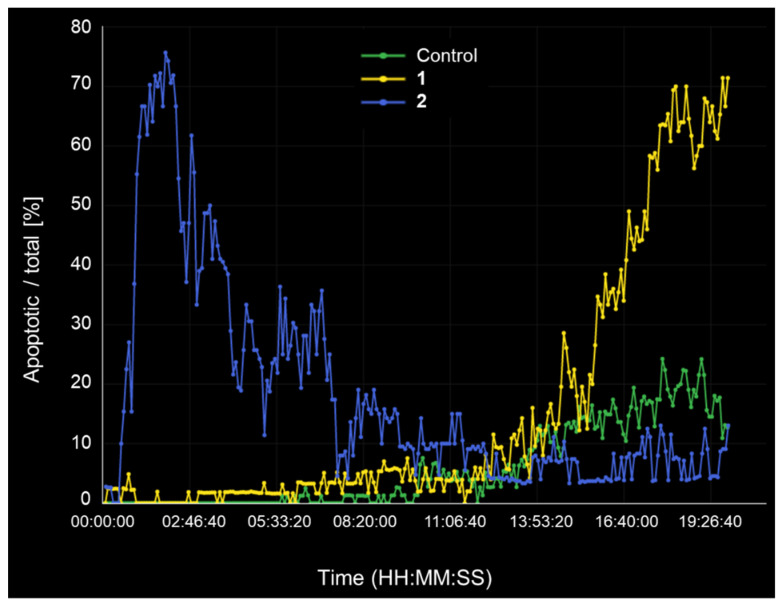
Label-free continuous live-cell imaging study on HeLa cells. Apoptosis kinetics obtained with LCDA software based on refractive indexes resulting from CX-A observation over time. Total exposure time 20 h. Green: untreated cells. Yellow: **1** (80 μM). Blue: **2** (50 μM).

The effects of compounds **1** and **2** on HeLa cells were studied in detail using label-free continuous live-cell imaging (Figure 9). Live-cell imaging eases the study of phenotypic changes at single cell level and enables checking the effects at continuous time points, changing the concept of dose–response relationship at fixed times. Label-free studies avoid confounding effects induced by the dyes. HeLa cells were exposed to compound **1** and compound **2** separately for 20 h. The selected doses, 80 and 50 µM were chosen based on the TGI values against HeLa cells, i.e., 82 ± 4.5 and 46 ± 0.3 µM, respectively. Images were recorded every 5 min. Continuous live-cell imaging enabled observing cell death progressively, distinguishing several apoptotic hallmarks (Supplementary material Videos S1 and S2). The LCDA analysis allowed monitoring of apoptosis kinetics (Figure 9), i.e., the percentage of apoptotic cells at every time interval. Figure 9 depicts how diterpene **2** (blue line) can induce death in HeLa cells earlier than coumarin **1** (yellow line). Thus, both compounds might have different modes of action, and most probably, they will have different cellular targets. The LC_50_ values for compounds **1**–**2** against HeLa cells are >100 and 69 ± 27 µM, respectively. We speculate that this might explain the observed differences in apoptosis kinetics.

## 4. Conclusions

For the first time, the antioxidant activity, the hypotensive effect, enzymatic inhibitory, and the antiproliferative potential have been studied, complemented with the full UHPLC phenolic fingerprinting of *G. pinifolium* Phil. Through UHPLC-PDA-Orbitrap-MS, it was possible to detect 26 metabolites. Besides, this is the first research on the inhibitory activity (against cholinesterase and tyrosinase) and antiproliferative screening of this shrub and its main components in the specified group of cancer cell lines. Enzymatic activity results showed a potent inhibition. Furthermore, the extracts plus pure compounds showed antiproliferative activity and relaxant effect in the rat aorta. This study highlights the potential of *G. pinifolium* extracts to become a product with nutritional and health-promoting properties with potentiality in chronic noncommunicable diseases. It is still necessary to elucidate which are the minor bioactive compounds that are responsible for the activities studied and to develop bioassay-guided fractionation with *G. pinifolium* extracts for isolation and identification to correlate the minor compounds to these interesting bioactivities.

## Figures and Tables

**Table 1:** Identification of phenolic compounds by HESI orbitrap HR-MS of *Gypothamnium pinifolium* extracts.

Peak #	Retention Time (min)	UV Max	Tentative Identification	Elemental Composition [M-H]	Measured Mass (m/z)	Theoretical Mass (m/z)	Accuracy (δ ppm)	MS^n^ Ions (δ ppm)
1	10.68	-	Diferulic acid	C_20_H_17_O_8_^−^	385.08936	385.07776	4.1	--
2	14.39	-	Vanillin	C_8_H_8_O_3_^−^	151.03960	151.03495	4.2	--
3	18.57	-	Camphoric acid	C_10_H_15_O_4_^−^	199.09649	199.09676	1.4	--
4	20.72	266	Jacareubin	C_18_H_13_O_6_^−^	325.06863	325.06798	2.1	--
5	20.89	266	6-Hydroxy-aphyllodenticulide	C_19_H_21_O_4_^−^	313.14478	313.14344	4.3	269.15475
6	21.05	266	4-Hydroxy-aphyllodenticulide	C_19_H_21_O_4_^−^	313.14490	313.14344	4.7	269.15468
7	21.26	271	6,12′-Dihydroxy-2-*nor*-1,2-secolycoserone	C_24_H_31_O_6_^−^	415.21307	415.21152	3.8	343.11902, 315.16046
8	21.44	275	Pinoresinol	C_20_H_21_O_6_^−^	357.13477	357.13326	4.2	338.35379
9	21.62	264	3,4-Divanillyltetrahydrofuran	C_20_H_23_O_5_^−^	343.15549	343.15400	4.3	336.19431
10	21.88	282	12′-Hydroxylycoserone	C_25_H_29_O_6_^−^	425.19739	425.19587	3.5	325.18454, 125.09155, 225.20520
11	22.05	270	Gypothamniol	C_25_H_29_O_5_^−^	409.20239	409.20095	3.5	315.1643
12	22.25	255	*Ent*-labda-8,13-*E*-diene-15-ol	C_19_H_22_O_6_^−^	289.25639	289.25635	1.2	--
13	22.54	264	12′-Hydroxy-2-*nor*-1,2-secolycoserone	C_24_H_31_O_5_^−^	399.21817	399.21660	3.9	331.53766, 243.17702
14	22.91	273	7′,8′,10′-Trihydro-lycoserone	C_25_H_27_O_4_^−^	391.19122	391.19039	2.1	299.12909
15	23.02	266	Aphyllodenticulide *	C_19_H_21_O_4_^−^	313.14389	313.14344	1.5	269.15681
16	23.29	263	8-*epi*-gypothamniol	C_25_H_29_O_5_^−^	409.20245	409.20095	3.7	325.18430
17	23.78	273	10′,11′-Dehydro-lycoserone	C_25_H_27_O_5_^−^	407.18686	407.18978	3.8	407.18683
18	23.98	272	6,11′-Dihydroxy -lycoserone	C_25_H_29_O_5_^−^	441.19209	441.19078	3.1	331.86105, 320.18945
19	24.24	279	Lycoserone (1′-b-H -lycoserone)	C_25_H_29_O_5_^−^	409.20245	409.20095	3.7	392.59332, 307.17134
20	24.93	282	Cyclolycoserone	C_25_H_31_O_5_^−^	411.21790	411.21660	3.1	396.61743, 352.60742, 334.64893, 331.85318
21	25.09	282	*Epi*-cyclolycoserone (1′-b-H -cyclolycoserone)	C_25_H_31_O_5_^−^	411.21793	411.21660	3.2	396.61743, 352.60742, 334.64893, 331.85318
22	25.72	282	10′-Hydroxylycoserone	C_25_H_29_O_6_^−^	425.19742	425.19587	3.6	382.48810, 265.14792
23	26.56	282	11′-Hydroxylycoserone	C_25_H_29_O_6_^−^	425.19736	425.19587	3.5	405.61282, 399.21716, 377.62582, 307.19763
24	27.07	274	*Epi*-lycoserone (1′-a-H -lycoserone)	C_25_H_29_O_5_^−^	409.20197	409.20095	2.5	334.62164
25	27.60	276	9-Reduced-10′-11′ dehydro-lycoserone	C_25_H_33_O_5_^−^	413.23389	413.23225	4.0	321.24384, 317.00323
26	29.09	271	2-*nor*-1,2-secolycoserone *	C_24_H_31_O_4_^−^	383.22314	383.22169	3.8	241.12337, 141.0156, 160.84164, 107.05024

* Identified by co-spiking using isolated compounds.

**Table 2:** Total phenolic and antioxidant activity of *G. pinifolium*.

Assay	TPC ^A^	TFC ^B^	DPPH ^C^	ABTS ^C^	FRAP ^D^	ORAC ^E^
*n*-hexane extract	517.4 ± 12.5	72.3 ± 3.70	269.55 ± 2.06	411.95 ± 6.37	347.12 ± 1.15	287.3 ± 1.54
EtOAc extract	538.4 ± 4.70	465.8 ± 27.5	140.23 ± 1.85	112.30 ± 0.46	267.19 ± 1.36	256.82 ± 1.67
BHT	-	-	25.09 ± 0.55	-	-	-
Trolox	-	-	-	2.33 ± 0.11	-	-

All values were expressed as Mean ± SD (*n* = 3). ^A^ Expressed in mg gallic acid equivalent per g of dry extract. ^B^ Expressed in µmol Trolox equivalent per g of dry extract. ^C^ IC_50_ in μg per mL. ^D^ Expressed in μmol Trolox equivalent per gram of dry extract. ^E^ Expressed in μmol Trolox equivalents per gram of dry extract.

**Table 3:** Enzymatic inhibitory activity (IC_50_, in µg/mL) of *G. pinifolium*.

Assay	AChE Inhibition	BChE Inhibition	Tyrosinase Inhibition
*n*-hexane extract	4.58 ± 0.04	23.44 ± 0.03	9.25 ± 0.15
Ethyl acetate extract	6.43 ± 0.03	33.25 ± 0.02	12.32 ± 0.21
2-*nor*-1,2-secolycoserone (1)	1.21 ± 0.03	11.23 ± 0.02	3.23 ± 0.12
*ent*-labda-8,13-*E*-diene-15-ol (2)	5.45 ± 0.02	18.34 ± 0.08	17.25 ± 0.18
Galantamine	0.55 ± 0.03	3.82 ± 0.02	-
Kojic acid	-	-	0.76 ± 0.05
Quercetin	-	-	-

All values were expressed as means ± SD (*n* = 3). AChE, Acetylcholinesterase; BChE, Butyrylcholinesterase. Values in the same column are significantly different (at *p* < 0.05).

**Table 4:** Binding energies (kcal/mol) were obtained from docking experiments of major metabolites from the *G. pinifolium* n-hexane and ethyl acetate extracts, as well as the known inhibitors galantamine and kojic acid over acetylcholinesterase (*Tc*AChE) butyrylcholinesterase (*h*BChE) and tyrosinase.

Compound	Acetylcholinesterase Binding Energy	Butyrylcholinesterase Binding Energy	Tyrosinase Binding Energy
**Cyclolycoserone**	−11.973	−9.724	−7.047
8-epi-gypothaminol	−12.683	−8.442	−5.079
2-*nor*-1,2-secolycoserone	−13.396	−9.738	−5.663
*Ent*-labda-8,13-*E*-diene-15-ol	−10.097	−8.206	−5.495
6-Hydroxyaphyllodenticulide	−10.983	−8.615	−5.237
Galantamine	−12.989	−7.125	-
Kojic acid	-	-	−6.050

**Table 5:** Antiproliferative activity (GI_50_, in µM) against human solid tumor cell lines.

Compound	A549	HBL-100	HeLa	SW1573	T-47D	WiDr
**1**	23 ± 7.2	31 ± 0.3	21 ± 4.5	25 ± 5.6	30 ± 0.1	36 ± 4.1
**2**	4.5 ± 0.1	10 ± 1.3	4.8 ± 0.3	5.4 ± 0.6	5.1 ± 0.3	9.0 ± 3.4
Cisplatin	4.9 ± 0.2	1.9 ± 0.2	1.8 ± 0.5	2.7 ± 0.4	17 ± 3.3	23 ± 4.3

All values were expressed as means ± SEM (*n* = 3). Cisplatin was used as a reference compound.

## Data Availability

All of the data is contained within the article and the supplementary materials.

## Supplementary Materials

The following supporting information can be downloaded at: https://www.mdpi.com/article/10.3390/antiox11122313/s1, Figure S1: Full MS spectra and structures of coumarins compounds. A: 2-nor-1,2-secolycoserone, B: Lycoserone, C: 6,11′-dihydroxy-1′ H-lycoserone, and D: 9-reduced-10′,11′-dehydro-1′-H-lycoserone, Figure S2: 1H NMR experiment for compound **1**, Figure S3: 13C NMR experiment for compound **1**, Figure S4: DEPT 13C NMR experiment for compound **1**, Figure S5: 1H NMR experiment for compound **2**, Figure S6: 13C NMR experiment for compound **2**, Figure S7: DEPT 13C NMR experiment for compound **2**, Figure S8: Molecular structure of the 2-nor-1,2-secolycoserone and ent-labda-8,13-E-diene-15-ol, Table S1: 1H NMR (500 MHz, CDCl_3_) experimental data for compound **1**, Table S2: 13C NMR (126 MHz, CDCl3) experimental data for compound **1**, Table S3: 1H NMR (500 MHz, CDCl3) experimental data for compound **2**, Table S4: 13C NMR (126 MHz, CDCl3) experimental data for compound **2**, Video S1: Live-cell imaging of HeLa cells exposed to compound **1** (80 µM), Video S2: Live-cell imaging of HeLa cells exposed to compound **2** (50 µM).
