# Supplementary material for: Phenolic Fingerprinting and Bioactivity Profiling of Extracts and Isolated Compounds from Gypothamnium pinifolium Phil
Source: Antioxidants (Basel). 2022 Nov 22;11(12):2313. doi: 10.3390/antiox11122313 (PMC9774750; doi:10.3390/antiox11122313)
Supplement: Supplementary file 1 [file antioxidants-11-02313-s001.zip › antioxidants-1999741-supplementary .pdf]

Supplementary Material for the Article

Phenolic Fingerprinting and Bioactivity Profiling of Extracts and Isolated Compounds from *Gypothamnium pinifolium* Phil.

Ruth E. Barrientos[1], Elena Ibáñez[2], Adrián Puerta[3], José M. Padrón[3], Adrián Paredes[4,9], Fredi Cifuentes[5, 6], Javier Romero-Parra[7], Javier Palacios[8*], Jorge Borquez[9], and Mario J. Simirgiotis[1*]

Citation:

Received: date
Accepted: date
Published: date

Publisher's Note: MDPI stays neutral withregardtojurisdictionalclaims in published maps and institutionalaffiliations.

Copyright: © 2021 by the authors. Submittedforpossible open accesspublicationunderthetermsand conditionsoftheCreativeCommonsAttribution (CC BY) license (http://creativecommons.org/licenses/by/4.0/).

1 Instituto de Farmacia, Facultad de Ciencias, Universidad Austral de Chile, Campus Isla Teja, Valdivia, 5090000, Chile; ruth.barrientos@alumnos.uach.cl
2 Laboratory of Foodomics, Institute of Food Science Research, CIAL, CSIC, Nicolás Cabrera 9, Madrid, 28049, Spain; elena@ifi.csic.es
3 BioLab, Instituto Universitario de Bio-Orgánica Antonio González (IUBO-AG), Universidad de La Laguna, 38206 La Laguna, Spain; apuertaa@ull.es (A.P.); jmpadron@ull.es (J.M.P.)
4 Laboratorio de Química Biológica, Instituto Antofagasta, Universidad de Antofagasta, Antofagasta 1270300, Chile; adrian.paredes@uantof.cl
5. Laboratorio de Fisiología Experimental, Instituto Antofagasta, Universidad de Antofagasta, Antofagasta 1270300, Chile; fredi.cifuentes@uantof.cl
6. Departamento Biomédico, Facultad Ciencias de la Salud, Universidad de Antofagasta, Antofagasta, 1240000, Chile; fredi.cifuentes@uantof.cl
7. Departamento de Química Orgánica y Fisicoquímica, Facultad de Ciencias Químicas y Farmacéuticas, Universidad de Chile, Olivos 1007, Casilla 233, Santiago, Chile; javier.romero@ciq.uchile.cl
8 Laboratorio de Bioquímica Aplicada, Química y Farmacia, Facultad de Ciencias de la Salud, Universidad Arturo Prat, Iquique, 1110939; Chile; clpalaci@unap.cl
9 Departamento de Química, Facultad de Ciencias Básicas, Universidad de Antofagasta, Antofagasta, 1240000, Chile; jorge.borquez@uantof.cl
* Correspondence: mario.simirgiotis@gmail.com or mario.simirgiotis@uach.cl (M.J.S.); clpalaci@unap.cl (J.P.); Tel.: +56-63-63233257 (M.J.S.); +56-57-2526910 (J.P.)

Isolation Procedure

For the separation and isolation of two of the main compounds, adsorption chromatography with an open column was used, as well as a Chromatroton and columns coupled to a medium-pressure pump. First, the n-hexane extract was submitted to column chromatography (kieselgel 60 H, 500 g) to obtain 355 fractions which were grouped into five groups (A-E) according to TLC plates analysis (silica gel plates f254, mobile phase n-hexane:ethyl acetate 80:20 v:v, developed with p-anisaldehyde and heating), n-hexane and ethyl acetate were used as mobile phase in different proportions: 100% n-hexane, n-hexane:EtOAc 90:10 v:v, n-hexane:EtOAc 80:20 v:v, n-hexane:EtOAc 70:30 v:v, n-hexane:EtOAc 60:40 v:v, n-hexane:EtOAc 50:50 v:v. Fractions A (F1-F182) and B (F183-F233) were not used for isolation since they contained the lipid part of the extract. Fraction C (F234-F295) and D (F296-F334) contain compounds of intermediate polarity, including the two compounds of interest, while fraction E (F335-F355) contain more polar compounds. The C fraction (3.8 g) was used to isolate compounds 2-*nor*-1,2-secolycoserone (retention factor = 0.53) and *ent*-labda-8,13-*E*-diene-15-ol (retention factor = 0.44).

Fraction C (1.4 g) was further fractionated using a 4 mm disc with a flow rate of 8 mL/min on Chromatroton, employing n-hexane: EtOAc gradients. The following fractions were obtained: 32

fractions (F1-32) with 250 mL of 95:5 v:v; 19 fractions (F33-51) using 94:6 v:v; ; 6 fractions (F52-57) with 93:7 v:v; 6 fractions (F58-63) using 92:8 v:v; 6 fractions (F64-69) for 91:9 v:v; 5 fractions (F70-74); for 90:10 v:v; 6 fractions (F75-80) using 85:15 v:v; 6 fractions (F81-85)for 82:18 v:v; 6 fractions (F86-91) for 80:20 v:v; and finally 16 fractions (F92-107) using 100% EtOAc. Then, from the TLC analysis, it was decided to combine fractions 29 to 56 (550 mg), and two 2 mm disc were used on Chromatroton (with 200 mg of sample each one) with a flow rate of 5.2 mL/min, with a mobile phase of n-hexane EtOAc 80: 20 v:v. Finally, for the first 2 mm disc, 75 fractions were collected and *ent*-labda-8,13-*E*-diene-15-ol was separated from F41 to F56, then with the second disc 50 fractions were collected and the same compound was separated from F15 to F39.

For the isolation of 2-*nor*-1,2-secolycoserone from fraction C, medium pressure column chromatography was used (kiesegel 60 G) with flow rate of 7 mL/min. For the first colum, 100% n-hexane, 90:10 v:v n-hexane and EtOAc, and finally 80:20 n-hexane and EtOAc were employed. For the second column, the gradient used was 90:10 v:v then 80:20 v:v of n-hexane and ethyl acetate. Finally, to obtain the isolated compound, it was necessary to carry out the crystallization with n-hexane:ethyl acetate 9:1 v/v, as previously described [1].

LC Parameters and MS Parameters

Liquid chromatography was performed using a UHPLC C18 column (Acclaim, 150 × 4.6 mm ID, 2.5 μm; Thermo Fisher Scientific, Bremen, Germany) operated at 25 °C. The detection wavelengths were 280, 254, 330, and 354 nm, and photodiode array detector was set from 200 to 800 nm. Mobile phases were 1% formic acid aqueous solution (A) and acetonitrile 1% formic acid (B). The gradient program started at 5% B at zero time, then maintained 5% B for 5 min, then going to 30% B for 10 min, then maintaining 30% B for 15 min, then going to 70% B for 5 min, then maintaining 70% B for 10 min, and finally going back to initial conditions in 10 and 12 min for column equilibration before each injection. The flow rate was 1.00 mL/min, and the injection volume was 10 μL. Standards, n-hexane and ethyl acetate extracts dissolved in methanol were kept at 10 °C during storage in the autosampler. The HESI II and Orbitrap spectrometer parameters were optimized as previously reported [2]. Briefly, as follows: Sheath gas flow rate, 75 units; auxiliary gas unit flow rate, 20; capillary temperature, 400 °C; auxiliary gas heater temperature, 500 °C; spray voltage, 2500 V (for ESI−); and S lens, RF level 30. Full scan data in positive and negative were acquired at a resolving power of 70,000 FWHM at *m*/*z* 200. Scan range of *m*/*z* 100–1000; automatic gain control (AGC) was set at 3×10^6 and the injection time was set to 200 ms. The chromatographic system was coupled to MS with a source II heated electro-nebulization ionization probe (HESI II). Nitrogen gas carrier (purity >99.999%) was obtained from a Genius NM32LA (Peak Scientific, Billerica, MA, USA) generator and used as a collision and damping gas.

The mass calibration for Orbitrap was performed every day, to ensure the accuracy of an operating mass equal to 5 ppm. A mixture of taurocholic acid sodium salt, buspirone hydrochloride, and sodium dodecyl sulfate (Sigma-Aldrich, Darmstadt, Germany), plus Ultramark 1621 (Alpha Aezar, Stevensville, MI, USA), a fluorinated phosphazine solution, was used as a standard mixture. These compounds were dissolved in a mixture of acetic acid, acetonitrile, water, and methanol (Merck, Santiago, Chile), and infused using a Chemyx Fusion 100 (Thermo Fisher Scientific, Bremen, Germany) syringe pump every day. The Q-Exactive 2.0 SP 2, Xcalibur 2.3, and Trace Finder 3.2 (Thermo Fisher Scientific, Bremen, Germany) were used for UHPLC mass spectrometer control and data processing, respectively.

Docking studies

First, the geometries and partial charges of every compound shown in Figure 1 were fully optimized using the DFT/B3LYP method with a standard basis set 6-311G/+dp in Gaussian 09W software [3–5]. Energetic minimizations and protonation or deprotonation (if applicable), were carried out using the LigPrep tool in Maestro Schrödinger suite v.11.8 (Schrödinger, LLC) [6].

Crystallographic enzyme structures of Torpedo Californica acetylcholinesterase (TcAChE; PDBID: 1DX6 code [7]), human butyrylcholinesterase (hBChE; PDBID: 4BDS code [8]) and the *Agaricus bisporus* mushroom tyrosinase (tyrosinase; PDBID: 2Y9X code [9]) were downloaded from the Protein Data Bank RCSB PDB [10]. Enzyme optimizations were carried out using the ProteinPreparation Wizard available in Maestro software, where water molecules and ligands of thecrystallographic protein active sites were removed. In the same way, all polar hydrogen atoms at pH
= 7.4 were added. Appropriate ionization states for acid and basic amino acid residues were considered. The OPLS3e force field was used to minimize protein energy as well. The enclosing box size was set to a cube with sides of 26 Å length.

The centroid of selected residue was chosen based on the putative catalytic site of each enzyme, considering their known catalytic amino acids: Ser200 foe TcAChE [11,12], Ser 198 for hBChE [13,14], and His263 for tyrosinase [9,15,16]. The Glide Induced Fit Docking protocol has been used for the final couplings [17]. Compounds were punctuated by the Glide scoring function in the extra-precision mode (Glide XP; Schrödinger, LLC) [18] and were filtered on the basis of the best scores and best RMS values (less than 1 unit as a cutting criterion), in order to obtain the potential intermolecular interactions between compounds and the enzymes, as well as the binding mode and docking descriptors. The different complexes were visualized in a Visual Molecular Dynamics program (VMD) and Pymol [19].

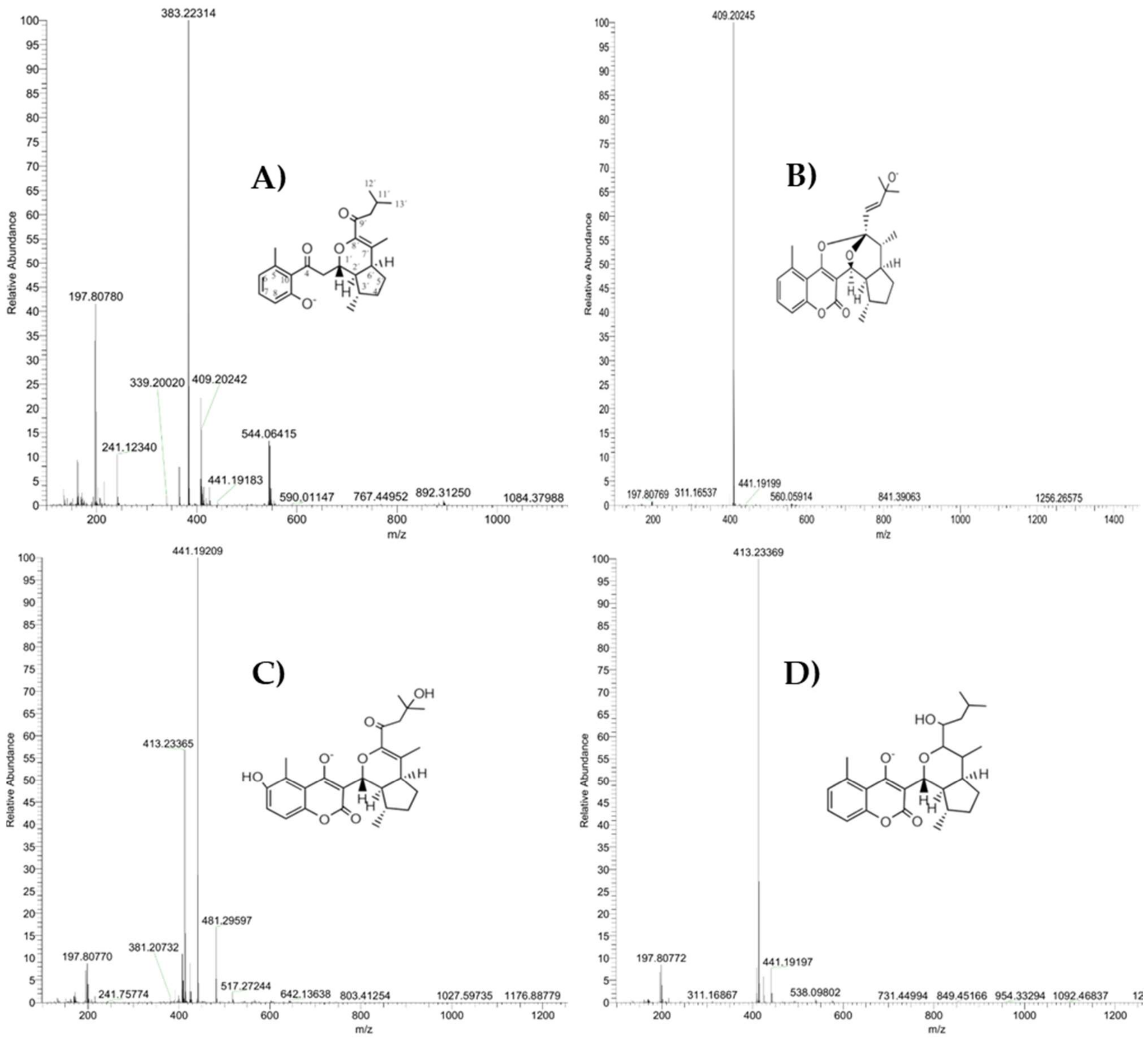

Figure S1. Full MS spectra and structures of coumarins compounds. A: 2-*nor*-1,2-secolycoserone, B: Lycoserone, C: 6,11′-dihydroxy-1′ H-lycoserone, and D : 9-reduced-10′,11′-dehydro-1′-H-lycoserone.

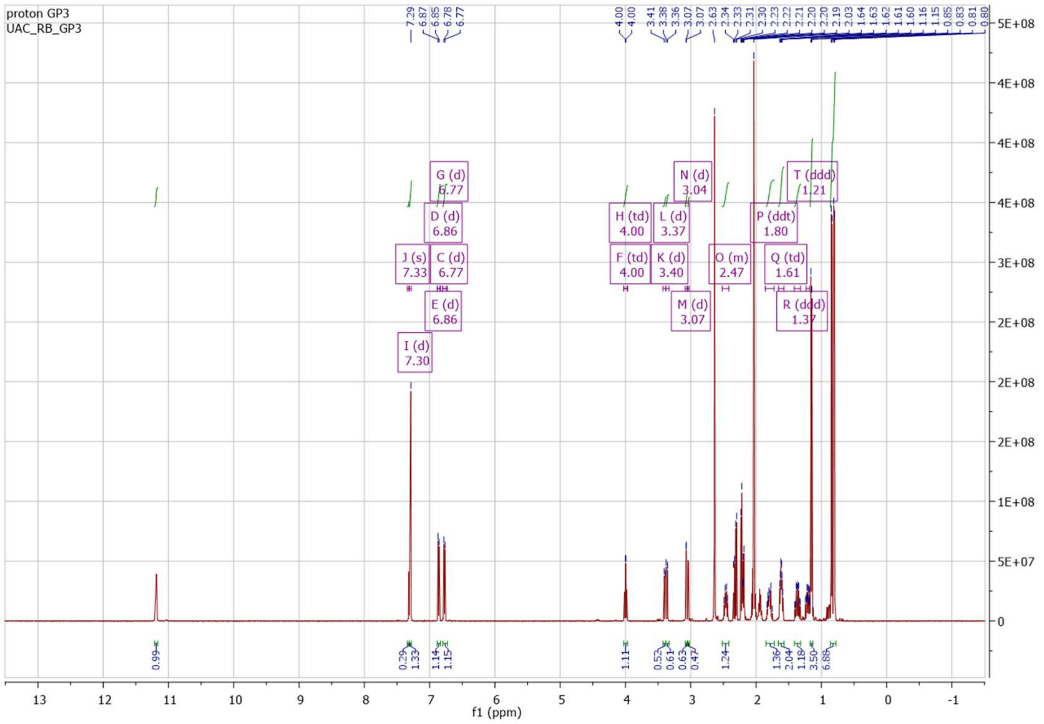

Figure S2. ^{1}H NMR experiment for compound 1.

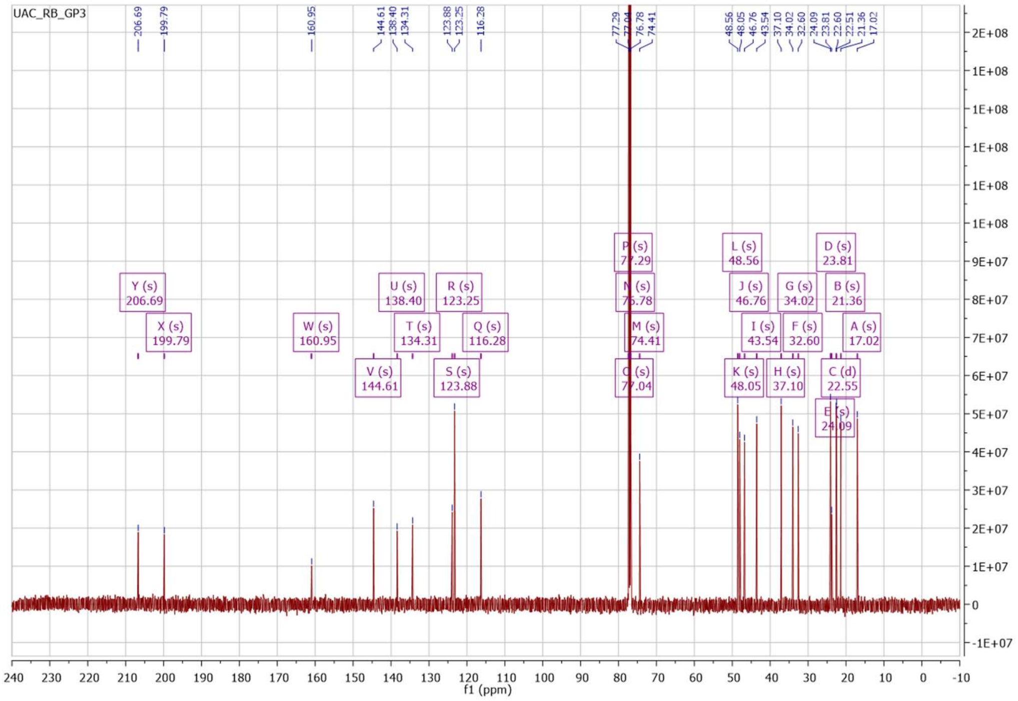

Figure S3. ^{13}C NMR experiment for compound 1.

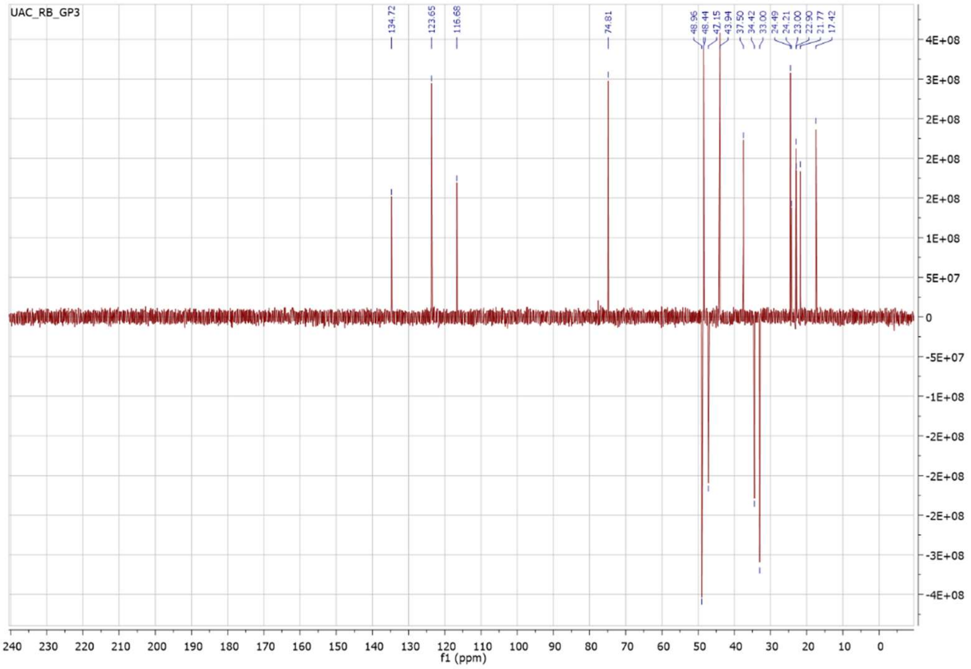

Figure S4. DEPT ^{13}C NMR experiment for compound 1.

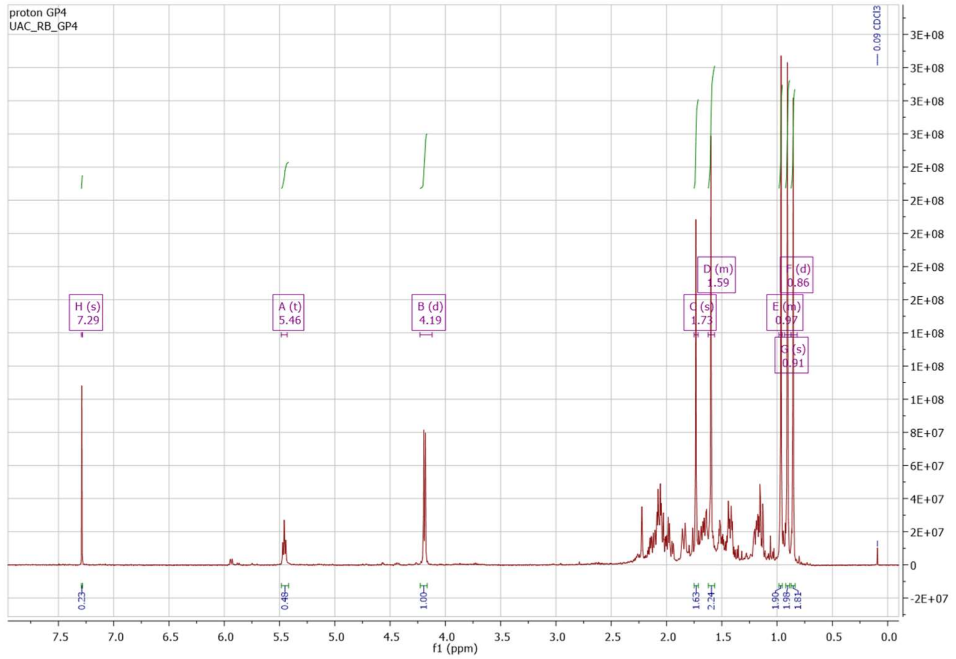

Figure S5. ^{1}H NMR experiment for compound 2.

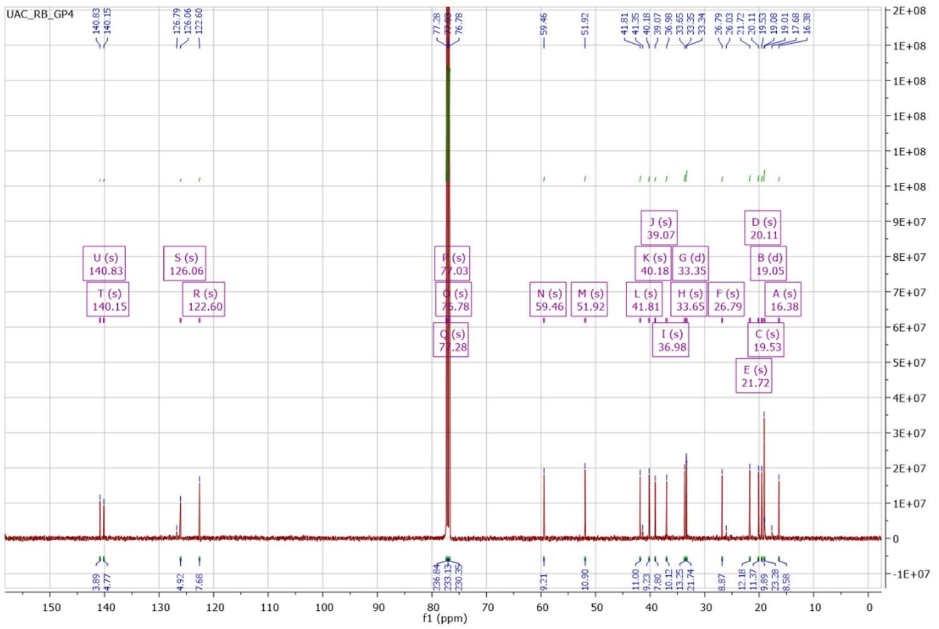

Figure S6. ^{13}C NMR experiment for compound 2.

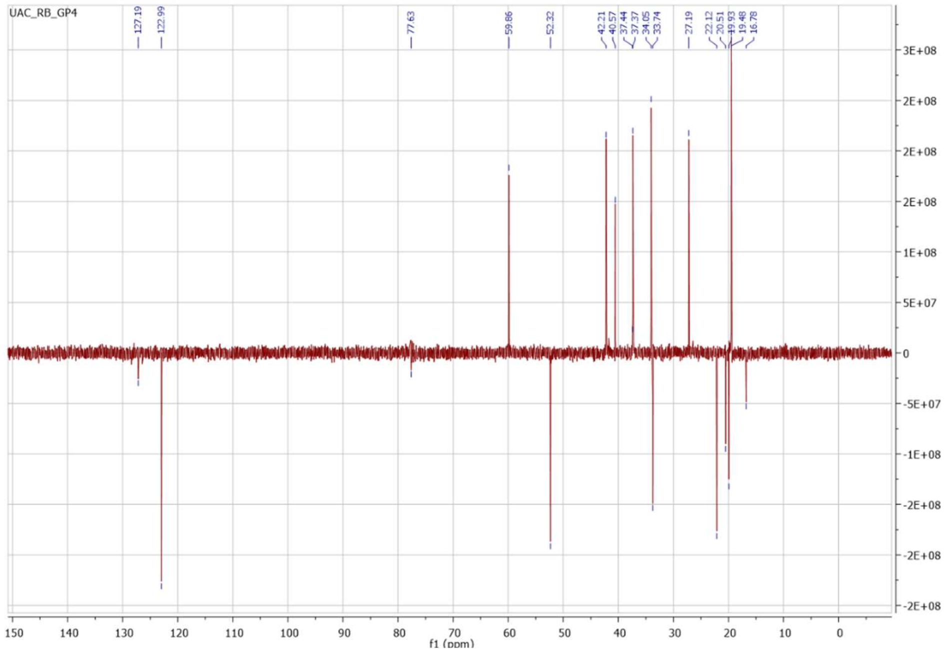

Figure S7. DEPT ^{13}C NMR experiment for compound 2.

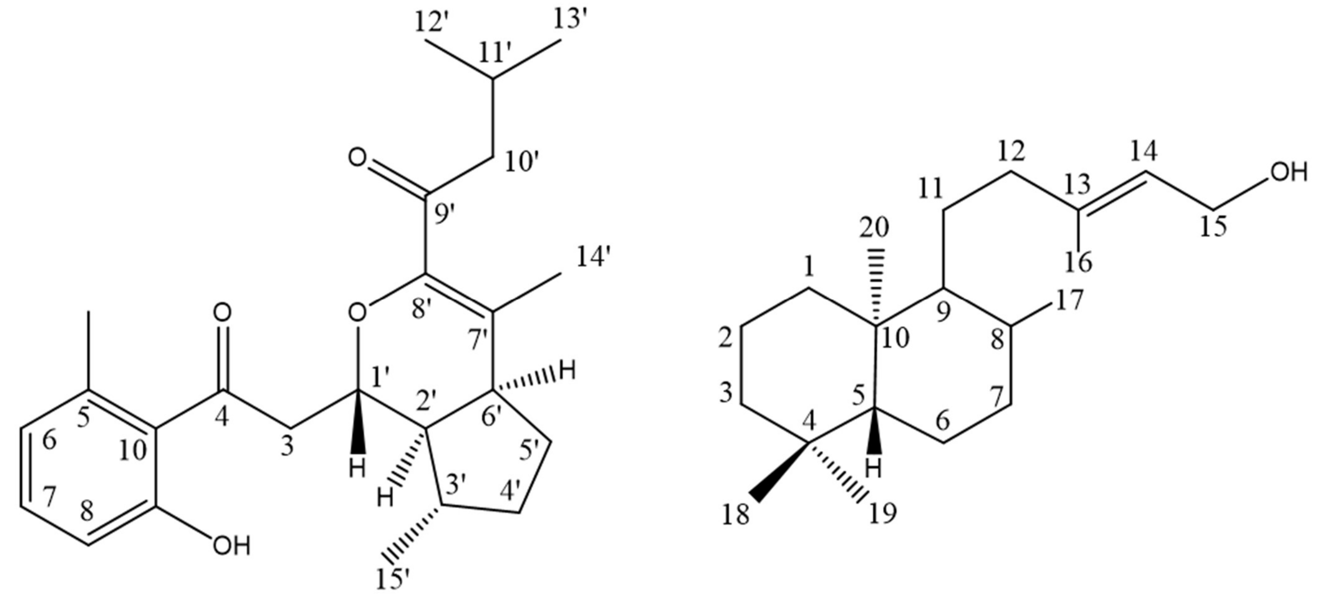

Figure S8. Molecular structure of the 2-*nor*-1,2-secolycoserone and *ent*-labda-8,13-*E*-diene-15-ol.

Supplementary table S1. ^{1}H NMR (500 MHz, $CDCl_3$) experimental data for compound 1.

^{1}H NMR	Chemical shift, δ_H (ppm)
7-H	7.30 (d, *J* = 8.1 Hz)
8-H	6.86 (d, *J*= 8.3 Hz)
6-H	6.77 (d, *J* = 7.4 Hz)
1′-H	4.00 (td, *J* = 9.8 Hz, 2.5 Hz)
	3.40 (d, *J* = 9.8 Hz)
	3.04 (d, *J* = 2.5 Hz)
11-H	2.52-2.42 (m)
6′-H	2.45 (*J* = 5.6 Hz)
10_1′-H	2.34
10_2′-H	2.23
5_1′-H	2.20
14′-H	2.03
3′-H	1.80 (ddt)
2′-H	1.61 (td, *J* =9.2 Hz, 5.2 Hz)
5_2′-H	1.37 (ddd, *J* = 23.3 Hz, 11.9 Hz, 6.4 Hz)
4_2′-H	1.21 (ddd, *J* = 12.2 Hz, 6.1 Hz, 3.5 Hz)
15′-H	1.15
12′-H	0.83
13′-H	0.80

Supplementary table S2. ^{13}C NMR (126 MHz, $CDCl_3$) experimental data for compound 1.

^{13}C NMR	Chemical shift, δ_C (ppm)
3	46.76
4	206.69
5	138.40
6	123.25
7	134.31
8	116.28
9	160.95
10	123.88
11	21.36
1′	74.41
2′	48.05
3′	37.10
4′	34.02
5′	32.60
6′	43.54
7′	144.61
8′	123.25
9′	199.79
10′	48.56
11′	23.81
12′	22.6
13′	22.55
14′	17.02
15′	24.09

Supplementary table S3. ^{1}H NMR (500 MHz, $CDCl_3$) experimental data for compound 2.

^{1}H NMR	Chemical shift, δ_H (ppm)
14-H	5.38 (t, *J* = 6.6 Hz, 1H)
15_1-H, 15_2-H	4.62 (d, *J* = 7.1 Hz, 2H)
17-H	1.76 (s, 3H)
16-H	1.62 – 1.57 (m, 3H)
18-H	0.99 – 0.94 (m, 3H)
20-H	0.92 (d, *J* = 9.7 Hz, 3H)
19-H	0.86 (d, *J* = 6.9 Hz, 3H)

Supplementary table S4. ^{13}C NMR (126 MHz, $CDCl_3$) experimental data for compound 2.

**^{13}C NMR**	**Chemical shift, δ_C (ppm)**
1	33.35
2	26.79
3	36.98
4	36.98
5	51.92
6	19.05
7	39.07
8	126.06
9	140.15
10	40.18
11	41.81
12	41.81
13	140.83
14	122.60
15	59.46
16	19.05
17	20.11
18	33.65
19	21.72
20	16.38

References

1. Simirgiotis, M.J.; Bórquez, J.; Neves-Vieira, M.; Brito, I.; Alfaro-Lira, S.; Winterhalter, P.; Echiburú-Chau, C.; Jerz, G.; Cárdenas, A. Fast isolation of cytotoxic compounds from the native Chilean species Gypothamnium pinifolium Phil. collected in the Atacama Desert, northern Chile. *Ind. Crops Prod.* **2015**, *76*, doi:10.1016/j.indcrop.2015.06.033.

2. Larrazábal-Fuentes, M.J.; Fernández-Galleguillos, C.; Palma-Ramírez, J.; Romero-Parra, J.; Sepúlveda, K.; Galetovic, A.; González, J.; Paredes, A.; Bórquez, J.; Simirgiotis, M.J.; et al. Chemical Profiling, Antioxidant, Anticholinesterase, and Antiprotozoal Potentials of Artemisia copa Phil. (Asteraceae). *Front. Pharmacol.* **2020**, *11*, 1, doi:10.3389/fphar.2020.594174.

3. Petersson, G.A.; Bennett, A.; Tensfeldt, T.G.; Al-Laham, M.A.; Shirley, W.A.; Mantzaris, J. A complete basis set model chemistry. I. The total energies of closed-shell atoms and hydrides of the first-row elements. *J. Chem. Phys.* **1988**, *89*, 2193–2218, doi:10.1063/1.455064.

4. McLean, A.D.; Chandler, G.S. Contracted Gaussian basis sets for molecular calculations. I. Second row atoms, Z=11-18. *J. Chem. Phys.* **1980**, *72*, 5639–5648, doi:10.1063/1.438980.

5. Frisch, A. Gaussian 09W Reference 2009.

6. Release, S. (2018) 2 Maestro, version 11.8. Schrdinger, LLC, New York. - References - Scientific Research Publishing.

7. Greenblatt, H.M.; Kryger, G.; Lewis, T.; Silman, I.; Sussman, J.L. Structure of acetylcholinesterase complexed with (-)-galanthamine at 2.3 Å resolution. *FEBS Lett.* **1999**, *463*, 321–326, doi:10.1016/S0014-5793(99)01637-3.

8. Nachon, F.; Carletti, E.; Ronco, C.; Trovaslet, M.; Nicolet, Y.; Jean, L.; Renard, P.Y. Crystal structures of human cholinesterases in complex with huprine W and tacrine: Elements of specificity for anti-Alzheimer's drugs targeting acetyl- and butyryl-cholinesterase. *Biochem. J.* **2013**, *453*, 393–399, doi:10.1042/BJ20130013.

9. Ismaya, W.T.; Rozeboom, H.J.; Weijn, A.; Mes, J.J.; Fusetti, F.; Wichers, H.J.; Dijkstra, B.W. Crystal structure of agaricus bisporus mushroom tyrosinase: Identity of the tetramer subunits and interaction with tropolone. *Biochemistry* **2011**, *50*, 5477–5486, doi:10.1021/bi200395t.

10. Berman, H.M.; Westbrook, J.; Feng, Z.; Gilliland, G.; Bhat, T.N.; Weissig, H.; Shindyalov, I.N.; Bourne, P.E. The Protein Data Bank. *Nucleic Acids Res.* **2000**, *28*, 235–242, doi:10.1038/s41577-020-00473-z.

11. Silman, I.; Harel, M.; Axelsen, P.; Raves, M.; Sussman, J.L. Three-dimensional structures of acetylcholinesterase and of its complexes with anticholinesterase

agents. In Proceedings of the Biochemical Society Transactions; Portland Press Ltd, 1994; Vol. 22, pp. 745–749.

12. Sussman, J.L.; Harel, M.; Frolow, F.; Oefner, C.; Goldman, A.; Toker, L.; Silman, I. Atomic structure of acetylcholinesterase from Torpedo californica: a prototypic acetylcholine-binding protein. *Science (80-.).* **1991**, *253*, 872–879.

13. Nicolet, Y.; Lockridge, O.; Masson, P.; Fontecilla-Camps, J.C.; Nachon, F. Crystal Structure of Human Butyrylcholinesterase and of Its Complexes with Substrate and Products. *J. Biol. Chem.* **2003**, *278*, 41141–41147, doi:10.1074/jbc.M210241200.

14. Tallini, L.R.; Bastida, J.; Cortes, N.; Osorio, E.H.; Theoduloz, C.; Schmeda-Hirschmann, G. Cholinesterase inhibition activity, alkaloid profiling and molecular docking of chilean rhodophiala (Amaryllidaceae). *Molecules* **2018**, *23*, doi:10.3390/molecules23071532.

15. da Silva, A.P.; Silva, N. de F.; Andrade, E.H.A.; Gratieri, T.; Setzer, W.N.; Maia, J.G.S.; da Silva, J.K.R. Tyrosinase inhibitory activity, molecular docking studies and antioxidant potential of chemotypes of Lippia origanoides (Verbenaceae) essential oils. *PLoS One* **2017**, *12*, e0175598, doi:10.1371/journal.pone.0175598.

16. Chen, J.; Ye, Y.; Ran, M.; Li, Q.; Ruan, Z.; Jin, N. Inhibition of Tyrosinase by Mercury Chloride: Spectroscopic and Docking Studies. *Front. Pharmacol.* **2020**, *11*, 81, doi:10.3389/fphar.2020.00081.

17. Sherman, W.; Day, T.; Jacobson, M.P.; Friesner, R.A.; Farid, R. Novel procedure for modeling ligand/receptor induced fit effects. *J. Med. Chem.* **2006**, *49*, 534–553, doi:10.1021/jm050540c.

18. Friesner, R.A.; Murphy, R.B.; Repasky, M.P.; Frye, L.L.; Greenwood, J.R.; Halgren, T.A.; Sanschagrin, P.C.; Mainz, D.T. Extra precision glide: Docking and scoring incorporating a model of hydrophobic enclosure for protein-ligand complexes. *J. Med. Chem.* **2006**, *49*, 6177–6196, doi:10.1021/jm051256o.

19. DeLano, W.L. The PyMOL molecular graphics system.
